# Chimeric Drug Design with a Noncharged Carrier for Mitochondrial Delivery

**DOI:** 10.3390/pharmaceutics13020254

**Published:** 2021-02-12

**Authors:** Consuelo Ripoll, Pilar Herrero-Foncubierta, Virginia Puente-Muñoz, M. Carmen Gonzalez-Garcia, Delia Miguel, Sandra Resa, Jose M. Paredes, Maria J. Ruedas-Rama, Emilio Garcia-Fernandez, Mar Roldan, Susana Rocha, Herlinde De Keersmaecker, Johan Hofkens, Miguel Martin, Juan M. Cuerva, Angel Orte

**Affiliations:** 1 Departamento de Fisicoquimica, Unidad de Excelencia de Química Aplicada a Biomedicina y Medioambiente, Facultad de Farmacia, Universidad de Granada, Campus Cartuja, 18071 Granada, Spain; consueloripoll@ugr.es (C.R.); pilarhf@ugr.es (P.H-F.); vpuente@ugr.es (V.P.-M.); mcarmeng@ugr.es (M.C.G.-G.); dmalvarez@ugr.es (D.M.); jmparedes@ugr.es (J.M.P.); mjruedas@ugr.es (M.J.R.-R); emiliogf@ugr.es (E.G.-F); 2 Departamento de Quimica Organica, Unidad de Excelencia de Química Aplicada a Biomedicina y Medioambiente, Facultad de Ciencias, Universidad de Granada, Campus Fuentenueva, 18071 Granada, Spain; sra@ugr.es (S.R.); jmcuerva@ugr.es (J.M.C.); 3 GENYO, Pfizer-University of Granada-Junta de Andalucía Centre for Genomics and Oncological Research. Avda. Ilustracion 114. PTS, 18016 Granada, Spain; miguelmartin@ugr.es (M.M.); mar.roldan@genyo.es (M.R.); 4 Department of Chemistry, K. U. Leuven, Celestijnenlaan 200F, B-3001 Heverlee, Belgium; susana.rocha@kuleuven.be (S.R.); herlinde.dekeersmaecker@kuleuven.be (H.D.K.); johan.hofkens@kuleuven.be (J.H.)

**Keywords:** antitumor agents, fluorescence lifetime imaging, medicinal chemistry, metabolic drug, mitochondrial carrier

## Abstract

Recently, it was proposed that the thiophene ring is capable of promoting mitochondrial accumulation when linked to fluorescent markers. As a noncharged group, thiophene presents several advantages from a synthetic point of view, making it easier to incorporate such a side moiety into different molecules. Herein, we confirm the general applicability of the thiophene group as a mitochondrial carrier for drugs and fluorescent markers based on a new concept of nonprotonable, noncharged transporter. We implemented this concept in a medicinal chemistry application by developing an antitumor, metabolic chimeric drug based on the pyruvate dehydrogenase kinase (PDHK) inhibitor dichloroacetate (DCA). The promising features of the thiophene moiety as a noncharged carrier for targeting mitochondria may represent a starting point for the design of new metabolism-targeting drugs.

## 1. Introduction

Mitochondria are essential organelles for cellular metabolism; therefore, understanding their function is critical for biologists and biochemists [1]. Importantly, cellular metabolism, through mitochondrial activity, plays a central role in many physiological alterations and diseases. Alterations in mitochondrial metabolism play a crucial role in cancer [2,3]. The anabolic pathways derived from the mitochondrial oxidative metabolism, such as tricarboxylic acid cycle (TCA)-derived biosynthetic reactions, are significantly essential to sustain high proliferative rates in many cancer types [4]. Nevertheless, other cancer cells display an apparent independence on mitochondrial oxidative metabolism, thus fundamentally relying on cytosolic biosynthetic pathways derived from glycolytic intermediates [5,6,7]. Within this context, targeting the mitochondria for either visualization or sensing depends on the existence of different carriers capable of delivering the intended compound inside this organelle [8,9].

Mitochondrial visualization using fluorescent probes has been extensively studied and has yielded high- and super-resolution images of this organelle [10,11,12]. Moreover, fluorescent sensors that target mitochondria have been used to determine real-time chemical and physiological information [13,14,15,16], and specific drug delivery to this organelle has been accomplished to enhance drug activity [17,18,19]. The working mechanism of existing carriers is based on two different properties of the mitochondrial membrane: the negative membrane potential and selective use of the mitochondrial protein import machinery [20]. The latter implies the use of characteristic oligopeptide sequences that must be included in the probe [21]. The former depends on the incorporation of a lipophilic cation, usually a phosphonium, ammonium or pyridinium salt, in the structure of the fluorophore or drug [22,23,24,25]. In fact, one of the most commonly employed mitochondrial delivery carriers is the triphenylphosphonium cation (TPP), a bulky, positively charged group [25]. Recently, it has been shown that increasing the lipophilic volume of the carrier using methylated phenyl radicals in TPPs further enhances mitochondrial accumulation [18,26]. Cationic dyes [27,28] and delocalized lipophilic cations [29] are also known to efficiently gather in such organelle. Nevertheless, although this strategy is widely used, it has some drawbacks. The first of them is related to the inherent synthetic difficulties associated with salts, which hamper the use of standard purification/synthetic procedures in an organic chemistry lab, therefore implying that these ionic functionalities must be introduced in the last synthetic steps. Thus for example, liquid-liquid extraction with organic solvents, a general procedure to isolate most of the drugs, is based on that some reagents, solvents or by-products are ideally displaced to the water phase, in which cationic species would be also soluble preventing this type of purification. Likewise, the most used chromatographic techniques do not allow the separation of this kind of compounds due to its ionic nature. All of these limit the structures to which these mitochondria carriers can be coupled. The second important drawback is related to alterations that lipophilic cations cause to the mitochondrial function. Several authors have pointed out that these structures are able to reduce mitochondrial membrane potential (ΔΨ_m_). During diverse stimulations or fixation with chemical agents, these dyes can easily leak out due to decrease in ΔΨ_m_, and cannot work effectively [14,30]. Moreover, deleterious effects over the electron transport chain and mitochondrial respiration have been reported to be caused by cationic dyes, normally used to measure ΔΨ_m_ [28], and mitochondria staining fluorescent probes [31].

**Scheme 1:**
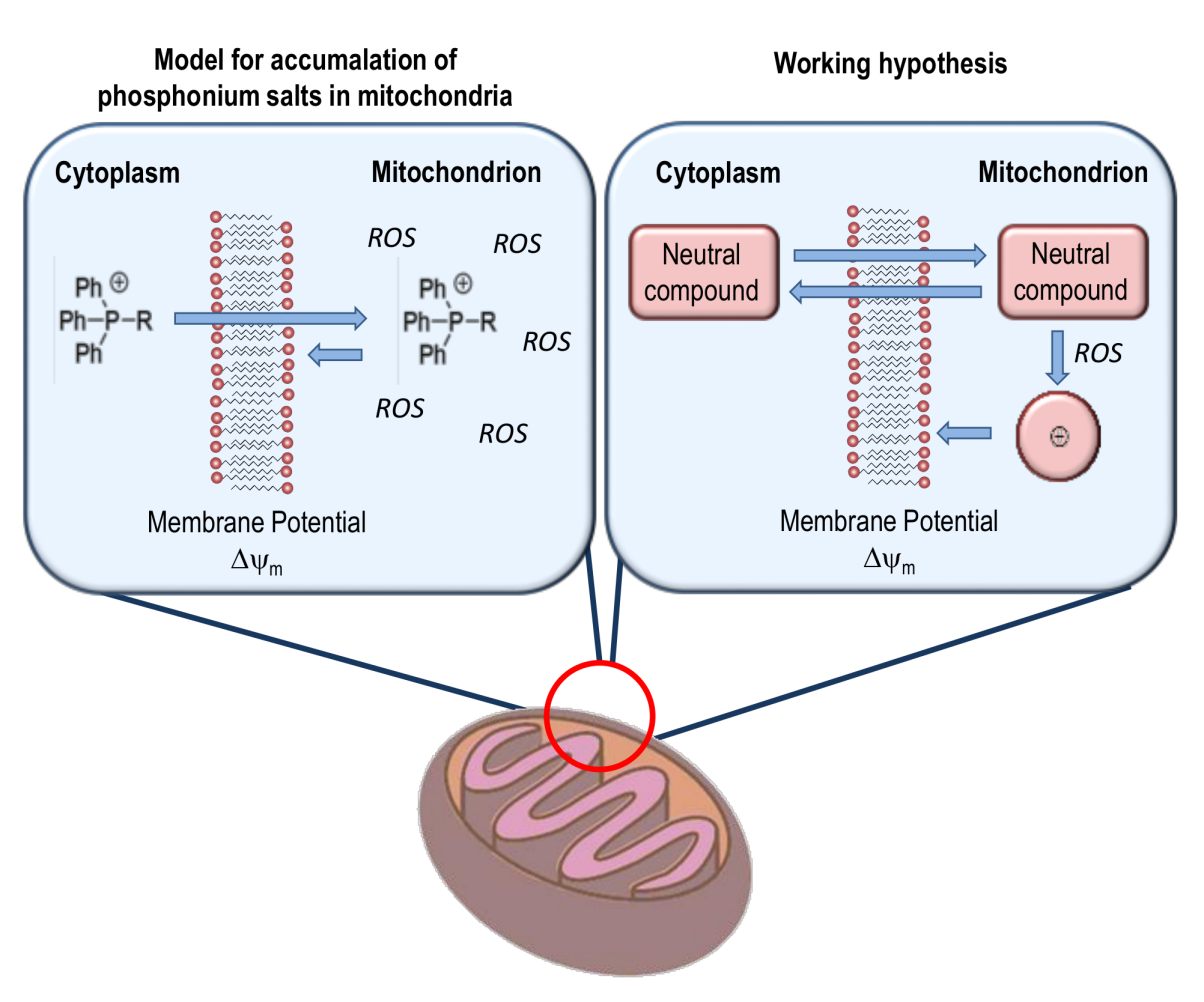
Comparison of traditional mitochondrial carriers (**left**) and our hypothesized noncharged carriers oxidized inside the mitochondria (**right**).

Development of a nonprotonable, noncharged and simple mitochondrial carrier could represent a new paradigm applicable not only to fluorescent probes but also to the selective transport of therapeutic drugs to mitochondria, as highlighted by Xu and Xu in a recent review on mitochondria-targeting fluorescent sensors [32]. From a synthetic point of view, drug modification with noncharged carriers clearly represents an improvement over traditional approaches in terms of the simplicity of chemical reactions, purification steps, and subsequent characterization by spectroscopic techniques. Bearing this in mind, we aimed to develop very simple noncharged mitochondrial carriers that are practically unknown. The working hypothesis is that species with a partial positive charge are directly generated and trapped in the interior of the mitochondrial matrix from suitable nonprotonable, noncharged species. Mitochondria are rich in reactive oxygen species (ROS) [33], and basic electron pairs can be oxidized in such environments. A neutral carrier could be transformed into a partially charged carrier by oxidation of a basic electron pair in its structure, fostering accumulation inside the organelle. In our hypothesis, a neutral carrier diffuses into the cytoplasm and enters the mitochondrial membrane, becoming trapped in the mitochondrial matrix by an oxidation reaction (Scheme 1). During the progress of our research, a similar mechanism to this proposal was reported by Reshetnikov and colleagues [17] based on the oxidation of ferrocene to ferrocenium cations, and by Abelha et al. [34], who employed oxidation of the TPP moiety. Another potential candidate for oxidation-driven mitochondrial accumulation is pyridine, a very stable aromatic substrate with a basic pair that can be oxidized to yield well-known pyridine oxides [35,36]. Unfortunately, the basicity of such an electron pair is high enough for interaction with acidic lysosomes, preventing entry into the mitochondria. Hence, we focused our attention on the electron pair of thiophene, which is essentially not basic under physiological conditions. However, thiophenes can be oxidized by oxygen-based oxidants [37], e.g., H_2_O_2_, as well as by cytochrome P450 in vivo [38,39], to yield thiophene oxides, dioxides and epoxides. Indeed, thiophene carriers exhibit enhanced mitochondrial accumulation when linked to acridone fluorescent markers, especially when designed for fluorescence lifetime imaging microscopy (FLIM) [40]. In this previous work, although *N*-(3-hydroxypropyl)-4-methoxy-acridone accumulated in the cell nucleus, since acridone dyes are excellent DNA binding agents [41], the addition of a terminal thiophene ring resulted in the preferential delivery of the fluorescent dye to the mitochondria instead of the nuclei [40]. Importantly, mitochondrial delivery of a noncharged, thiophene-containing acridone dye cannot be predicted by current quantitative structure-activity relation (QSAR) algorithms [42]. Therefore, confirmation of the general applicability of thiophene as a mitochondrial carrier is still lacking.

**Chart 1:**
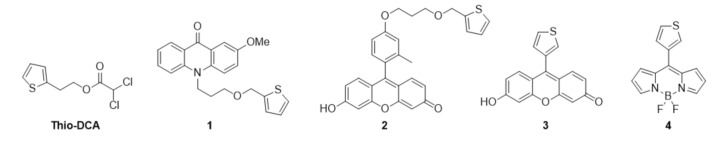
DCA-based drug Thio-DCA and thiophene-containing fluorescent dyes (acridone **1**, xanthene **2**–**3**, and BODIPY **4**) synthesized in this work.

Herein, we tested this new neutral mitochondrial carrier in a medicinal chemistry application by designing a thiophene-containing chimeric drug with potential anticancer activity after confirming the power of the thiophene ring to achieve mitochondrial delivery. One of the key targets in the regulation of cancer metabolism is pyruvate dehydrogenase complex (PDHC) [43], which catalyzes the key reaction to activate the TCA cycle. PDHC exhibits anomalously low activity in proliferative tumors that are resistant to conventional chemotherapies. This low pyruvate dehydrogenase (PDH) activity is caused by hyperactive pyruvate dehydrogenase kinase (PDHK), which phosphorylates PDH. In this context, therapies aiming to reactivate PDH would have an important impact, as they may decrease cellular metabolism to normal levels, circumventing the adaptive measures of tumor cells. With this in mind, mitochondrial accumulation of a PDHK inhibitor may lead to enhanced efficacy of metabolic treatment. Dichloroacetate (DCA) is a known PDHK inhibitor that has been tested in clinical trials, although its effectiveness has been questioned primarily because of the adverse side effects and toxicity due to the high doses needed to achieve antitumor activity [44]. Hence, we designed a very simple chimera of this PDHK inhibitor by merging DCA with a thiophene ring via an acetyl linker (Thio-DCA, Chart 1), and tested its efficacy against four different breast cancer cell lines that had shown resistance to DCA treatment, MDA-MB-468, MDA-MB-231, SKBR3, and MCF7, each with different metabolic features. It is relevant to work with all these cell lines due to their different cellular and molecular features and responses to chemotherapies and treatments [45].

## 2. Results

### 2.1. Synthesis of Thiophene-Modified Fluorescent Markers

In previous work, we showed that simple acridone-based fluorescent probe **1**, containing a thiophene side group (Chart 1), exhibited enhanced mitochondrial accumulation compared to its thiophene-lacking counterpart [40]. Acridone derivatives are known to be very good DNA intercalating agents and therefore usually accumulate in the nucleus [41]. They are also known to have very long fluorescence lifetimes, τ, which make them very suitable for FLIM imaging [46,47,48]. In fact, probe **1** permits the quantitative determination of microenvironment dipolarity inside mitochondria using the excellent sensing capabilities of the acridone moiety [40].

Encouraged by these results, we determined the performance of the thiophene group as a general mitochondrial carrier for use in medicinal chemistry applications after its incorporation into other fluorescent compounds with different chemical natures, polarities and lipophilic characteristics (Chart 1). Apart from acridone **1**, we synthesized a xanthene derivative with the thiophene group included on a flexible chain (**2**), a xanthene derivative with the thiophene incorporated in the central ring (**3**) and a BODIPY derivative (**4**).

**Scheme 2:**
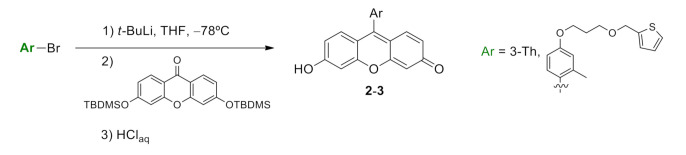
Synthetic route of compounds **2**–**3**.

Both xanthene derivatives, 6-hydroxy-9-(2-methyl-4-(3-(thiophen-2-ylmethoxy) propoxy)phenyl)-3*H*-xanthen-3-one (**2**) and thiophene-modified xanthene **3**, 6-hydroxy-9-(thiophen-3-yl)-3*H*-xanthen-3-one, were synthesized using a methodology developed by our group (Scheme 2) [49]. Thus, the addition of an organolithium derivative, obtained by bromine-lithium exchange of the corresponding aryl bromide, to the *tert*-butyldimethylsilyl ester (TBDMS)-protected xanthene moiety afforded compounds **2** and **3** after acid treatment. Compounds **2** and **3** were subsequently isolated as orange solids. Dye **2** is a derivative of 2-methyl-4-methoxy-phenyl xanthene, one of the so-called Tokyo Green dyes [50]. Finally, the *meso*-thiophene BODIPY, **4**, was prepared following a previously published route [51]. Further details regarding the synthetic protocol employed for each compound in Chart 1, as well as spectroscopic characterization (^1^H- and ^13^C-NMR spectral data) and mass spectrometry data, are described in the Experimental section and the Supplementary Materials (SM, Schemes S1–S6 and Figures S1–S16).

### 2.2. Thiophene as a General Mitochondrial Delivery Agent

The synthesized thiophene-containing fluorescent markers have different structural and physical features. Whereas acridone **1** and BODIPY **4** are pH-independent and neutral fluorophores, xanthene derivatives **2** and **3** exhibit a prototropic equilibrium between neutral and anionic forms upon deprotonation of the hydroxyl group in the xanthene moiety. Using UV-visible absorption spectroscopy, we confirmed that **1** and **4** exhibited pH-independent spectra (Figure S17 in the SM) and calculated the acid–base equilibrium constant, in terms of pK_a_, as 6.28 ± 0.06 and 6.39 ± 0.03 for **2** and **3** (Figure S18), respectively. This mild acidic behavior may already help these dyes localize to the mitochondria [42], but importantly, the presence of the neutral thiophene ring does not alter the acid–base properties or the charge of the dyes. Compound **2** maintained a high emission quantum yield (0.84 ± 0.02) owing to the 2-methyl substituent in the phenyl ring, which keeps it perpendicular to the xanthene plane, decreasing nonradiative deactivation. The perpendicular conformation of an aromatic substituent at the *meso*- position is a well-known requirement for high fluorescence emission efficiency in xanthene [50,52] and BODIPY [53,54] dyes. In contrast, the thiophene ring in xanthene **3** has free rotation, decreasing the fluorescence quantum yield down to 0.14 ± 0.01. In fact, fluorescence lifetime measurements exhibited faster deactivation and hence a lower quantum yield in low viscosity methanol:glycerine mixtures. At 20 °C, the fluorescence lifetime of **3** increased from 1.7 ± 0.1 ns in methanol (viscosity of 0.54 mPa·s) up to 2.54 ± 0.03 ns in a methanol:glycerine mixture of 2.02 mPa·s. This dependence indicates that rotation of the thiophene ring is involved in the deactivation pathway of **3**. Further details on the viscosity dependence of the fluorescence properties of **3** can be found in Figure S19.

**Figure 1:**
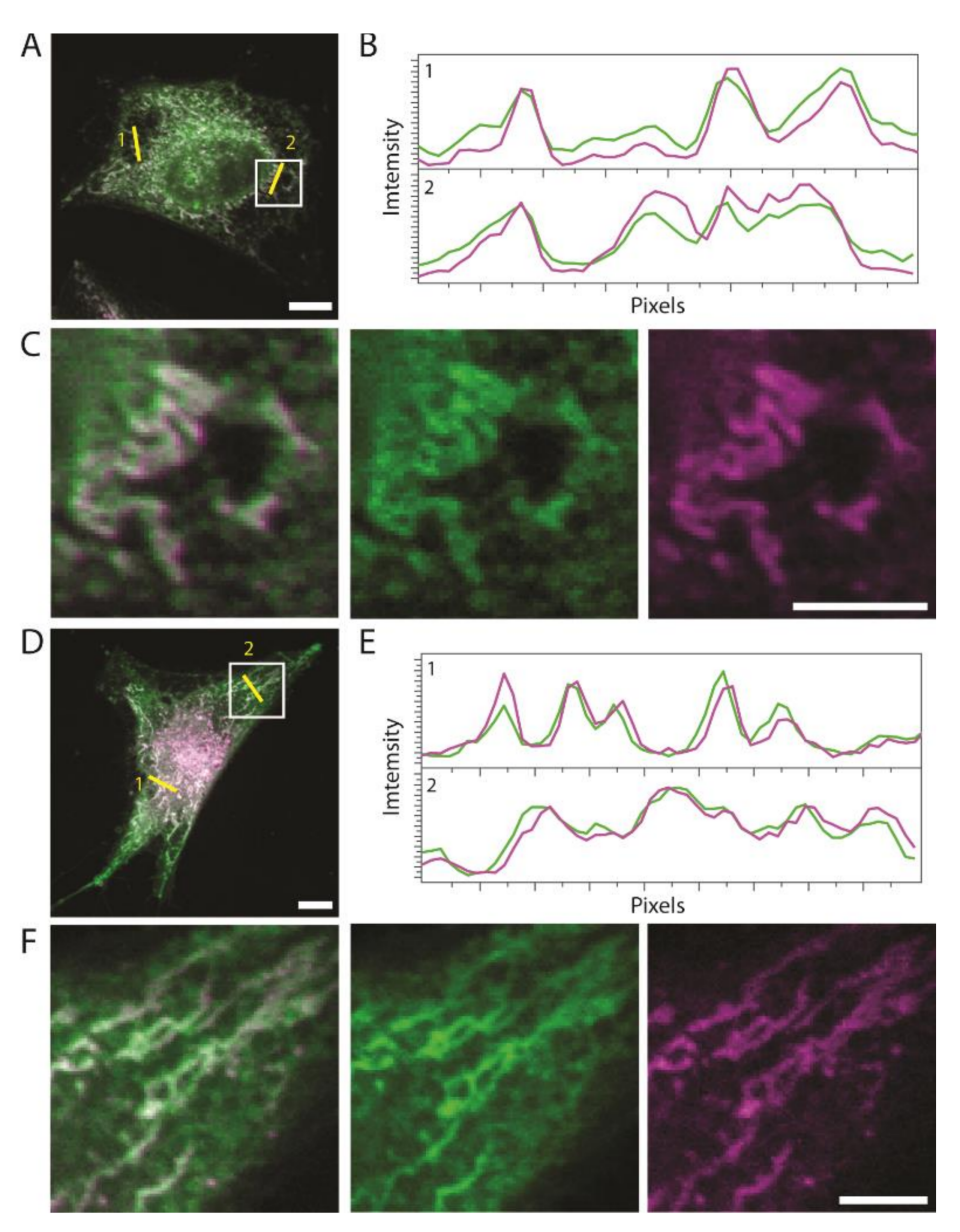
Dual-color confocal microscopy of **1** (**A**–**C**) and **2** (**D**–**F**) in formaldehyde-fixed HeLa cells. (**A**,**D**) Overlaid images of the dye (green) and MT (purple). Scale bars represent 10 μm. (**B**,**E**) Intensity profile traces extracted from the yellow lines in **A**,**D**. (**C**,**F**) Magnified image of the area in the white square, showing the two color channels split. Scale bars represent 5 μm.

Once we ensured that the dyes were photostable enough for imaging applications (Figure S20) and that the presence of the thiophene ring was not detrimental to the fluorescence properties of the dyes, we employed dual-color fluorescence microscopy for the simultaneous imaging of the dyes and mitochondria, which was traced in red using MitoTracker Deep Red (MT). By following the localization of the probe, we could easily detect the transport efficiency of the noncharged carrier to the mitochondria. Figure 1 shows assessment of the effective incorporation of dyes **1** and **2** into the mitochondria with great confidence. Additionally, we used dual-color FLIM microscopy to simultaneously examine the localization of the dyes and their emission kinetics through the fluorescence lifetime values (see the SM and Table S1 for experimental details). Figures S21–S29 in the SM show dual-color FLIM images of dyes **1**–**4** in live cells, demonstrating clear accumulation in the mitochondria. Nevertheless, a fraction of the dyes was also found in other cellular compartments, confirming that the accumulation is not perfectly specific, and further work may be required to achieve higher selectivity. In fact, the Pearson’s correlation coefficient (Table S2) averaged through at least five different images was 0.59 ± 0.10, 0.69 ± 0.07, 0.58 ± 0.12, and 0.67 ± 0.04 for **1**, **2**, **3**, and **4**, respectively. We also obtained the mutual Manders’ colocalization coefficients (MCC, Table S2) as a measure of the fraction of coincident pixels in each channel. The MCC values of colocalized dye with MT were 0.46 ± 0.14, 0.64 ± 0.14, 0.58 ± 0.15, and 0.46 ± 0.06 for **1**, **2**, **3**, and **4**, respectively. The MCC values for the MT channel, indicating the fraction of colocalized mitochondrial pixels, were 0.64 ± 0.10, 0.73 ± 0.14, 0.66 ± 0.12, and 0.36 ± 0.12 for **1**, **2**, **3**, and **4**, respectively. These values indicate a good level of colocalization, although accompanied by nonspecificity, as can be visually inspected. Notably, the dye that best localized in the mitochondria was **2**, in which the thiophene group is further apart from the chromophore moiety. In any case, these results indicate that thiophene may be a good carrier for mitochondrial drug delivery.

Accumulation driven by ΔΨ_m_ is usually related to positively charged lipophilic moieties, such as TPP [13]. Interestingly, dyes **1** and **4** are noncharged and neutral, yet they accumulated in the mitochondria. To evaluate whether ΔΨ_m_ is involved in the accumulation of the dyes in this organelle, we performed colocalization experiments of compound **1** and MT in the presence of BAM-15, a chemical known to disrupt ΔΨ_m_ but not affect the plasma membrane potential and thus prevent drastic consequences on cell viability [55]. After treatment with BAM-15, the mitochondria-tracking dye MT was expelled out of the mitochondria and accumulated in cytoplasmic vacuoles. Interestingly, a similar response was observed with the thiophene-labeled dye **1**, which in part appeared colocalized in the vacuoles with MT but also appeared more homogeneously throughout the cytoplasm (Figure S30). These experiments revealed that ΔΨ_m_ is directly involved in the accumulation of the thiophene-containing dyes in mitochondria. The mitochondrial targeting behavior was similar to that of the MT probe but based on a different carrying mechanism, as MT is a positively charged dye.

### 2.3. Thio-DCA, a PDHK Inhibitor with Enhanced Effectiveness

Having demonstrated the potential of the thiophene ring to enhance mitochondrial delivery of small organic molecules, we focused our attention on designing an effective mitochondrial chimeric drug, including an active agent and a specific subcellular delivery moiety [56]. The rational design of multifunctional imaging and therapeutic agents using a modular approach is an active field in current theranostics and drug discovery programs [57,58]. As previously described in the introduction, cancer cells rely extensively on the glycolytic pathway to obtain large amounts of metabolic intermediates, which are required as building blocks to sustain their high proliferative rate. In this metabolic reprogramming process, pyruvate is reduced at the end of glycolysis to lactate, and a minor proportion is transported into the mitochondria to be transformed to acetyl-CoA by PDH. This metabolic pathway has been recently targeted in drug screening strategies, with the hypothesis that molecules that could change the preferential metabolism of pyruvate by forcing its entry into mitochondrial metabolism may convey specific toxicity to cancer cells. For this work, we chose DCA as a known PDHK inhibitor capable of reactivating the PDH complex and, presumably, of switching cancer metabolism to foster mitochondria-regulated apoptosis. The main effects of DCA are a decrease in HIF-1α and Bcl-2 in neoplastic cells, followed by an increase in the expression of p-53 upregulated modulator of apoptosis, p-53 and caspases. As a consequence, negative modulation of the transcription of glucose transporter (GLUT) receptors occurs, attenuating the uptake of glucose in tumor cells [59]. Another effect of DCA on tumor cells is the increase in the generation of ROS, allowing the entry of the NADH generated in the Krebs cycle into complex I of the respiratory chain, reactivating PDH and favoring the remodeling of mitochondrial metabolism. This cascade facilitates the opening of the mitochondrial transition pore, which allows the release of proapoptotic mediators, such as cytochrome c and apoptosis-inducing factor, into the cytoplasmic space [60].

**Scheme 3:**
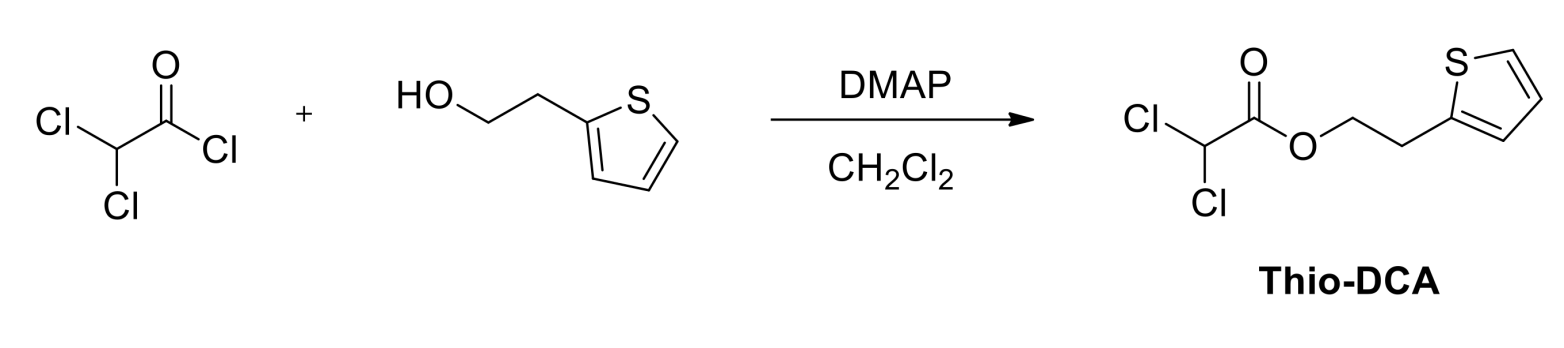
Synthesis of Thio-DCA.

Therefore, to enhance the effect of DCA, we synthesized thiophene-modified DCA, Thio-DCA, as depicted in Scheme 3. Further details on the synthesis and characterization can be found in the experimental section and the SM.

Regarding the cell lines employed in this work, we focused on breast cancer cells, since there exist different lines conventionally classified according to histological type, tumor grade, lymph node status and immune profile [45]: luminal A (cell line MCF7), luminal B (cell line ZR751), basal (cell line MDA-MB-468), claudin-low (cell line MDA-MB-231), and HER2^+^ (cell line SKBR3). MDA-MB-468 and MDA-MB-231 are commonly referred to as triple-negative breast cancer lines because of their negative expression of the estrogen receptor (ER), progesterone receptor (PR) and human epidermal growth factor receptor (HER2), presenting high invasiveness and poor prognostic outcome. In contrast, MCF7 and ZR751 are cell lines with a noninvasive low proliferation profile that results in good prognostic clinical outcomes. Finally, SKBR3 is a cell line that is HER2^+^ but negative for ER and PR. According to its response to treatment, SKBR3 cells show a response to the anti-HER2 therapy trastuzumab and a moderate response to chemotherapy. Therefore, it is relevant to work with all these cell lines due to their different cellular and molecular features and responses to chemotherapies and treatments [45]. Moreover, our recent results showed differences between these cell lines regarding intramitochondrial pH [15], resistance to transaminase inhibition, dependence on NAD^+^ availability, and sensitivity to alterations in glutamine metabolism [61].

In the first series of experiments, the impact of DCA-induced inhibition of PDHK on cell viability was measured in the aforementioned cell lines. For this purpose, cells were seeded with or without the addition of 10 mM DCA, and after 96 h incubation at 37 °C, cell viability was measured using the well-validated CellTiter Blue assay (see the Experimental section). These experiments permitted us to identify that the MCF7, SKBR3, MDA-MB-231 and MDA-MB-468 cell lines were quite resistant to DCA treatment, displaying cell viability values between 75–90%. Only the ZR751 cell line, with a decrease in cell viability up to 40%, was sensitive to DCA treatment [61]. Given that we wanted to directly compare the enhanced effect of Thio-DCA to the corresponding native DCA we decided not to use the sensitive cell line ZR751 in further experiments and thus only focused on the cell viability of DCA-resistant breast cancer lines upon incubation with Thio-DCA.

**Figure 2:**
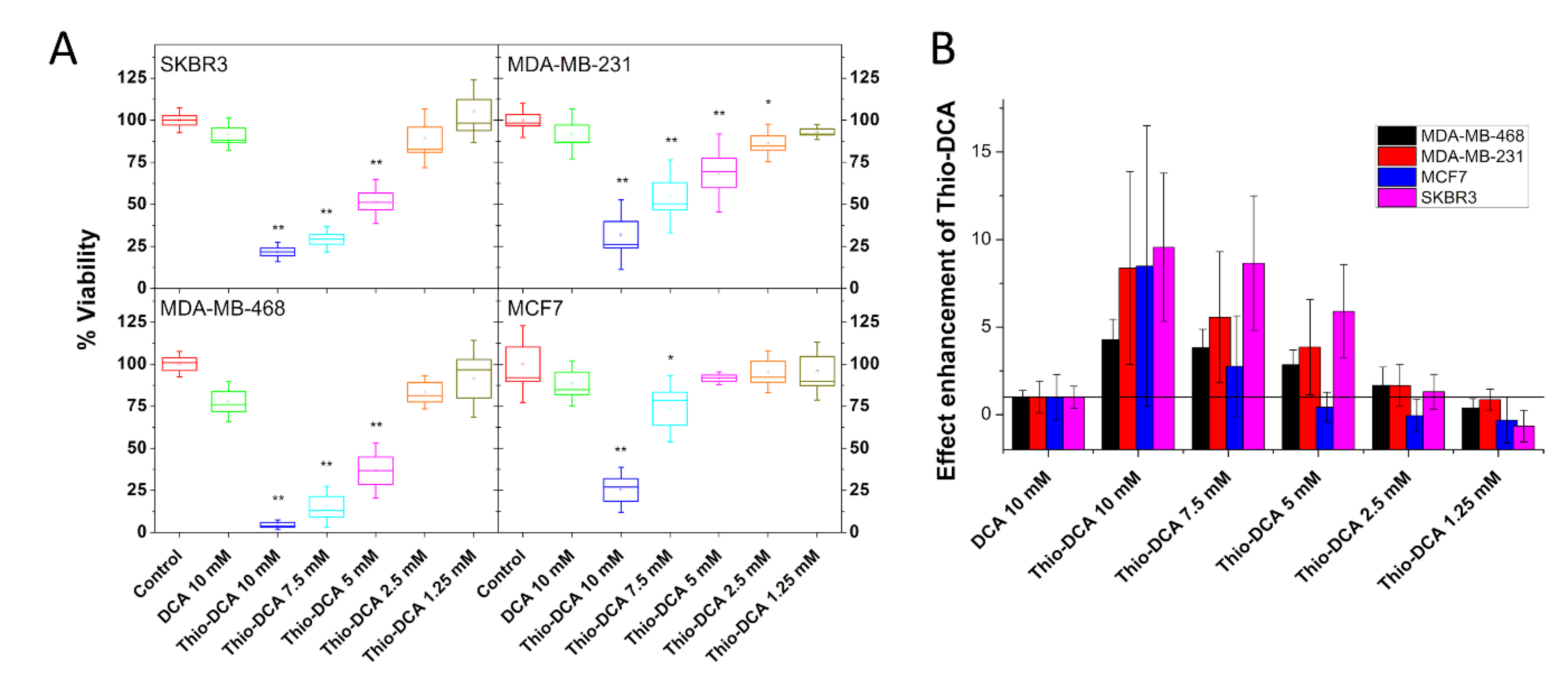
(**A**) Cell viability of SKBR3, MDA-MB-231, MDA-MB-468, and MCF7 breast cancer tumor cells treated with DCA and Thio-DCA at different doses. Squares indicate mean values, box size indicates the standard error of the mean, and whiskers represent the standard deviations. The marked populations were significantly different from the control with 99% (**) or 95% (*) confidence, as indicated by the Holm-Bonferroni and Holm-Sidak tests and the nonparametric Kolmogorov-Smirnov and Mann-Whitney tests. (**B**) Relative enhancement of the reduction in cell viability of Thio-DCA at different doses compared to that of 10 mM DCA.

Our experiments demonstrated that Thio-DCA presented enhanced, dosage-dependent antitumor activity compared to that presented by DCA for all four studied breast cancer cell lines (Figure 2). The IC_50_ values for Thio-DCA in the four cell lines were 5.9 ± 0.6, 7.8 ± 0.2, 4.0 ± 0.3, and 9.4 ± 0.1 mM for SKBR3, MDA-MB-231, MDA-MB-468, and MCF7, respectively. Thio-DCA at 10 mM exhibited between 4.3- and 9.6-fold increases in antiproliferation activity in the different breast cancer cell lines (Figure 2B) compared to DCA at the same concentration. The lowest value, a 4.3-fold increase, corresponded to MDA-MB-468 cells, mainly because 10 mM DCA caused a mild reduction in cell viability, down to 78 ± 6% viability. This makes the relative effect of Thio-DCA lower than that of DCA, but Thio-DCA at 10 mM in MDA-MB-468 cells presented the largest reduction in viability, down to 5 ± 1%, among all the tested treatments. For the other three cell lines, 10 mM DCA did not cause any significant reduction in cell viability, confirming the resistance of these cell lines to DCA treatment. Hence, the relative enhancement of the effect of 10 mM Thio-DCA was as large as 8.4–9.6-fold (Figure 2B). Lower concentrations of Thio-DCA still exhibited inhibition. For all cell lines, except MCF7, a Thio-DCA concentration of 5 mM resulted in a significant reduction in cell viability. The largest effect of Thio-DCA was found for the basal line MDA-MB-468. This cell line is characterized by a strong dependence on mitochondrial metabolism [61], and hence, our result is consistent with a larger effect of Thio-DCA due to enhanced mitochondrial delivery of the drug. In contrast, MCF7 cells exhibit glycolysis-dependent metabolism [61], and in turn, the effect of Thio-DCA less notable. The toxic effect of Thio-DCA on MDA-MB-468 cells supports other experimental observations currently in progress by our team that explain the toxicity of DCA treatments in cancer cells, which display a stronger dependency on mitochondrial metabolism. According to our ongoing observations, DCA causes a metabolic collapse of mitochondrial activity due to anaplerotic limitations in response to the DCA-driven activation of the tricarboxylic acid cycle.

The thiophene moiety is known to cause cellular toxicity when used at a high dosage due to oxidation by cytochrome P450 [38,39]. This toxicity may, however, be reduced by cellular oxidative stress response by glutathione [38,62]. Hence, we tested the potential toxicity associated with the thiophene moiety using 2-(3-thienyl)ethanol as the model precursor. 2-(3-thienyl)ethanol caused negligible effects on cell viability at concentrations lower than 5 mM. Reaching larger concentrations led to an IC_50_ value of 15 ± 1 mM (Figure S31). Therefore, although the thiophene moiety caused a reduction of cell viability at 10 mM, the effect was not as large as that exhibited by Thio–DCA.

## 3. Discussion

In seeking new, noncharged organelle-targeting carriers, we found that neutral acridone dyes underwent mitochondrial accumulation when modified with a thiophene ring [40]. Neutral organelle carriers are vastly desired from a synthetic point of view regarding their effect on intraorganelle ionic strength and membrane potentials. Logically, the subsequent step was to test the general applicability of this concept, as we did herein, synthesizing thiophene-containing dyes of different families, charged (xanthenes) and noncharged (acridone and BODIPY), and confirming the enhanced mitochondrial accumulation. We demonstrated the feasibility of the thiophene moiety as a noncharged, nonbasic carrier for targeting mitochondria. All fluorescent derivatives synthesized herein that contained a thiophene group showed good accumulation in mitochondria, although not completely specific in part due to the additional properties that the different fluorescent moieties impart to the molecule as a whole. In previous works, fluorescent dyes containing thiophene groups were reported to be delivered to the mitochondria [63,64,65], although accumulation in this organelle was justified by hydrophobicity and/or the presence of positive charges in ammonium side groups. For instance, TPP-modified polythiophenes, a construct in which polythiophene is the actual fluorophore, were shown to accumulate in mitochondria [66], although the driving force for mitochondrial delivery was assumed to be the TPP moiety. In contrast, we were able to define the different subcellular localization of dye **1** with respect to thiophene-less acridone [40], demonstrating and confirming herein the enhancement of mitochondrial delivery by the thiophene ring. An underlying active mechanism of mitochondrial accumulation driven by the thiophene ring is supported by the fact that well-known subcellular localization QSAR algorithms [42] cannot predict such organelle targeting for dye **1**. A recent algorithm based on machine learning trained with sets of literature data identified key structural motifs for subcellular localization among lysosomes, mitochondria, nucleus and plasma membrane [67]. This algorithm is included in the prediction tool admetSAR 2.0 [68]. The molecules in our work do not hold specific key motifs for mitochondrial localization. In contrast, the thiophene ring was identified by the authors to be a key motif for plasma membrane localization [67]. Interestingly, admetSAR 2.0 successfully predicted that all the molecules in our work would more likely localize in the mitochondria than in lysosomes, nuclei, or the plasma membrane. However, the algorithm also predicted mitochondrial localization for *N*-(3-hydroxypropyl)-4-methoxy-acridone, a compound that localizes in the cell nucleus [40]. Therefore, there is still room for improvement in machine-learning algorithms and QSAR models.

The most promising application, however, was the proof-of-concept of a chimeric drug with enhanced activity, promoted by thiophene-driven mitochondrial accumulation. Thio-DCA exhibited improved toxicity in breast cancer cell lines resistant to the thiophene-lacking counterpart DCA. This larger effect suggests that the inhibitor targets the locations on mitochondrial compartments where metabolic reactions occur. The concept of enhancing the activity of DCA with a mitochondrial carrier was previously reported by Pathak and colleagues, who prepared a chimera of positively charged TPP with three molecules of DCA, so-called Mito-DCA [24]. This molecule exhibited a much more potent effect than DCA alone in tumor cells, even at lower dosages than that reported by us with Thio-DCA. Nevertheless, Mito-DCA contains three DCA moieties per molecule and an overall positive charge that enhances mitochondrial depolarization [24]. In other examples, the anticancer power of haloacetates has been enhanced by incorporating DCA and other derivatives in phospholipid nanoparticles [69]. Increasing the local concentration of the inhibitor through the nanoparticle ensured efficient delivery. Although the doses used for Thio-DCA treatment are still high, they are lower than those employed in recent reports on the use of DCA, usually reaching up to 50 mM [44,70,71], and are similar to DCA concentrations used in sensitive cell lines [72]. The most important observation of our work is, however, that mitochondrial accumulation, promoted by a neutral carrier, enhanced the effect of the drug in several DCA-resistant cell lines. With this proof-of-concept, there is still plenty of room to improve drug efficacy by incorporating it in delivery nanoparticles [69] or supramolecular caging agents [73] and improving drug selectivity by reducing thiophene-associated toxicity by adding substitution radicals to the thiophene ring [38].

It is also informative to consider previous reports on anticancer drug design that involve thiophene cores. These drugs exhibited higher activity than previous inhibitors [74]. Likewise, thiophene-containing natural products, such as the bithiophenes arctinal and arctinol-b, have been shown to have antifungal and antimicrobial activity [75], as well as other cytotoxic effects on tumor cell lines [76]. Thiophene-modified coumarins also exhibit enhanced cytotoxicity towards cancer cell lines, whereas normal fibroblast cells are less affected [77]. In a recent report, Lisboa et al. described a chimeric drug of acridine andmodified thiophene with promising antitumoral activity [78]. We hypothesize that, in some cases, the primary cause of this activity may be inhibitor accumulation in the mitochondria, which is influenced by the thiophene core and enhances the inhibition mechanism by directly targeting where the metabolic reaction occurs.

Our current research lines aim to determine whether different subtypes of breast cancer cells display differential metabolic phenotypes [15,61]. Gaining knowledge about key metabolic features in breast cancer and their functional, molecular and genetic interrelationships could pave the way for a novel clinical classification, revealing potential therapeutic antimetabolic targets, and diagnostic approaches. The thiophene moiety as a noncharged neutral carrier for targeting mitochondria may represent a considerable advantage to overcome synthetic problems, paving the way to the rational design of new metabolism-targeted anticancer hybrid drugs [78,79]. The full potential of such drugs will be accomplished by incorporating additional substituents to the thiophene ring, aiming for improved selectivity in localization, activity and toxicity.

## 4. Materials and Methods

### 4.1. Synthesis Reactions of New Compounds

#### 4.1.1. General Details

All reagents and solvents (CH_2_Cl_2_, ethyl acetate (EtOAc), hexane, CH_3_CN, and methanol (MeOH)) were purchased from standard chemical suppliers and used without further purification. To ensure the dryness of tetrahydrofuran (THF), it was freshly distilled over Na/benzophenone. Anhydrous solvents (dimethylformamide (DMF), CH_2_Cl_2_ and diethyl ether (Et_2_O)) were purchased from standard suppliers. Thin-layer chromatography (TLC) was performed on aluminum-backed plates coated with silica gel 60 (230–240 mesh) with the F_254_ indicator. The spots were visualized with UV light (254 nm and 360 nm) and/or stained with phosphomolybdic acid (10% ethanol solution) and subsequent heating. All chromatographic purifications were performed with silica gel 60 (230–400 mesh). NMR spectra were measured at room temperature. ^1^H NMR spectra were recorded at 300, 400, or 500 MHz either in a Varian Direct Drive (Varian Inc., Palo Alto, CA, USA) or Bruker Avance NEO (Bruker BioSpin GmbH, Rheinstetten, Germany). Chemical shifts are reported in ppm using the residual solvent peak as a reference (CHCl_3_: δ = 7.26 ppm, CH_3_OH: δ = 3.31 ppm, and (CH_3_)_2_SO: δ = 2.50 ppm). Data are reported as follows: chemical shift, multiplicity (s: singlet, d: doublet, t: triplet, q: quartet, quint: quintuplet, m: multiplet, dd: doublet of doublets, dt: doublet of triplets, td: triplet of doublets, and bs: broad singlet), coupling constant (*J* in Hz) and integration. ^13^C NMR spectra were recorded at 75, 101, 126 or 151 MHz using broadband proton decoupling, and chemical shifts are reported in ppm using the residual solvent peaks as a reference (CHCl_3_: δ = 77.16 ppm, CH_3_OH: δ = 49.00 ppm, and (CH_3_)_2_SO: δ = 39.52 ppm). Carbon multiplicities were assigned by distortionless enhancement by polarization transfer (DEPT) techniques. High-resolution mass spectra (HRMS) were recorded by EI at 70 eV on a Micromass AutoSpec mass spectrometer (Waters Co., Milford, MA, USA) or by ESI on a Waters VG AutoSpec mass spectrometer (Waters Co.). For the synthesis of the compounds described herein, several precursors were required to be prepared, including 2-[3-(4-bromo-3-methyl-phenoxy)propoxymethyl]thiophene (precursor **VII,** Scheme S4 in the SM) and 2,7-di-[*tert*-butyldimethylsilyloxy]-xanthone (precursor **VIII**, Schemes S4 and S5 in the SM). Compounds **1** (Schemes S1–S3) and **4** (Scheme S6) were prepared as previously described, isolated as pure samples and showed NMR spectra identical to reported data [40,51]. Further details on the synthesis and characterization of all the precursors and compounds **1**–**4**, including ^1^H NMR and ^13^C NMR spectra (Figures S1–S16), are compiled in the SM.

#### 4.1.2. Synthesis of 6-hydroxy-9-(2-methyl-4-(3-(thiophen-2-ylmethoxy)propoxy)phenyl)-3H-xanthen-3-one (compound **2**, Scheme S4)

*t*-BuLi (1.7 M in hexane, 0.96 mL, 1.64 mmol) was added dropwise to a solution of compound **VII** (279 mg, 0.82 mmol) in freshly distilled THF (4 mL) under an Ar atmosphere at −78 °C. After keeping the reaction at that temperature for 20 min, a solution of compound **VIII** (187 mg, 0.41 mmol) in THF (2 mL) was slowly added. Then, the mixture was stirred at −78 °C for 15 min and allowed to reach room temperature. The reaction was monitored by TLC. After consumption of compound **VIII**, 10% HCl (1 mL) was added, promoting a color change from light yellow to orange. Finally, the solvent was removed, and the residue was purified by flash chromatography (SiO_2_, CH_2_Cl_2_/MeOH 9:1) to yield compound **2** (130 mg, 66%) as an orange solid. ^1^H NMR (400 MHz, MeOD) δ 7.36 (dd, *J* = 5.1, 1.2 Hz, 1H), 7.12 (d, *J* = 8.4 Hz, 1H), 7.08 (d, *J* = 9.7 Hz, 2H), 7.05–7.03 (m, 1H), 7.00 (d, *J* = 2.5 Hz, 1H), 6.9–6.94 (m, 2H), 6.68–6.64 (m, 4H), 4.72 (s, 2H), 4.17 (t, *J* = 6.2 Hz, 2H), 3.72 (t, *J* = 6.1 Hz, 2H), 2.09 (quint, *J* = 6.2 Hz, 2H), 2.02 (s, 3H). ^13^C NMR (101 MHz, MeOD) δ 179.0 (C), 161.5 (C), 159.7 (C), 156.5 (C), 142.5 (C), 138.9 (C), 132.3 (CH), 131.5 (CH), 127.59 (CH) 127.58 (CH), 126.8 (CH), 126.0 (C), 123.4 (CH), 117.6 (CH), 115.5 (C), 113.3 (CH), 104.5 (CH), 68.3 (CH_2_), 67.4 (CH_2_), 66.0 (CH_2_), 30.7 (CH_2_), 20.0 (CH_3_). HRMS (ESI): *m/z* [M+H]^+^ calculated for C_28_H_25_O_5_S: 473.1417 found: 473.1416.

#### 4.1.3. Synthesis of 6-hydroxy-9-(thiophen-3-yl)-3H-xanthen-3-one (compound **3**, Scheme S5)

*t*-BuLi (1.7 M in hexane, 0.66 mL, 1.12 mmol) was added dropwise to a solution of 3-iodothiophene (118 mg, 0.56 mmol) in freshly distilled THF (2 mL) under an Ar atmosphere at −50 °C. After keeping the reaction at that temperature for 20 min, a solution of compound **VIII** (128 mg, 0.28 mmol) in THF (2 mL) was slowly added. Then, the mixture was stirred at −50 °C for 15 min and allowed to reach room temperature. The reaction progress was monitored by TLC. After consumption of compound **VIII**, 10% HCl (1 mL) was added, promoting a color change from light yellow to orange. Finally, the solvent was removed, and the residue was purified by flash chromatography (SiO_2_, CH_2_Cl_2_/MeOH 8:2) to yield compound **3** (48 mg, 59%) as an orange solid. ^1^H NMR (500 MHz, DMSO-*d*_6_) δ 7.93–7.87 (m, 2H), 7.32 (dd, *J* = 4.7, 1.4 Hz, 1H), 7.22 (d, *J* = 9.3 Hz, 2H), 6.64 (bs, 4H). ^13^C NMR (126 MHz, DMSO-*d*_6_) δ 145.4 (C), 132.2 (C), 130.5 (CH), 129.3 (CH), 128.0 (CH), 127.7 (CH). Several carbons are not observed. HRMS (EI): *m/z* [M]^+^ calculated for C_17_H_10_O_3_S: 294.0351 found: 294.0339.

#### 4.1.4. Synthesis of Thio-DCA

4-dimethylaminopyridine (DMAP, 429 mg, 3.51 mmol) was added to a solution of 2-thiopheneethanol (0.26 mL, 2.34 mmol) in CH_2_Cl_2_ (5 mL). After 5 min, dichloroacetyl chloride (0.27 mL, 2.81 mmol) was added dropwise. The mixture was stirred at room temperature and monitored by TLC until consumption of starting materials occurred (5–10 min). Celite was then added, and the solvent was removed. The crude material was purified by flash chromatography (SiO_2_, hexane/EtOAc 9:1) to yield Thio-DCA (512 mg, 91%) as a light yellow liquid. ^1^H NMR and ^13^C NMR spectra are shown in the SM. ^1^H NMR (400 MHz, CDCl_3_) δ 7.19 (dd, *J* = 5.1, 1.2 Hz, 1H), 6.96 (dd, *J* = 5.1, 3.4 Hz, 1H), 6.90 (dd, *J* = 3.4, 1.2 Hz, 1H), 5.95 (s, 1H), 4.49 (t, *J* = 6.7 Hz, 2H), 3.25 (t, *J* = 6.7 Hz, 2H). ^13^C NMR (75 MHz, CDCl_3_) δ 164.5 (C), 138.7 (C), 127.2 (CH), 126.1 (CH), 124.5 (CH), 67.6 (CH_2_), 64.3 (CH), 29.0 (CH_2_). HRMS (ESI): *m/z* [M+Na]^+^ calculated for C_8_H_8_O_2_Cl_2_SNa: 260.9514 found: 260.9513.

### 4.2. Instrumentation

Absorption spectra of the different dyes in aqueous solutions were obtained on a Lambda 650 UV–visible spectrophotometer (PerkinElemer, Waltham, MA, USA). The steady-state fluorescence emission and excitation spectra were collected using a Jasco FP-8300 spectrofluorometer (Jasco, Tokyo, Japan). Time-resolved fluorescence decay traces were obtained on a FluoTime 200 SPT spectrofluorometer (PicoQuant GmbH, Berlin, Germany). The concentration of dyes in spectroscopic measurements was between 0.5 × 10^−6^ and 5 × 10^−6^ M.

Colocalization studies using dual-color confocal microscopy were performed on a Fluoview FV1000 laser scanning microscope (Olympus, Tokyo, Japan). For imaging compound **2**, compound **3** or MT, the sample was excited with a 405 nm, 488 nm or 635 nm laser line, respectively. Emission from compounds **2** (between 410 and 490 nm) and **3** (between 500 and 580 nm) was collected using a grating, whereas emission from MT was collected with a BA655-755 bandpass filter. Dual-color FLIM experiments were performed on a MicroTime 200 microscope (PicoQuant GmbH, Berlin, Germany) equipped with 440 nm, 470 nm, and 635 nm pulsed diode lasers as excitation sources. Emission from fluorophores **1**–**4** was separated from that of MT using a 600DCXR dichroic mirror. Different bandpass filters were used for the collection of the fluorescence of **1** (465/30), **2**–**4** (520/35) and MT (685/70). Further experimental settings for each instrument can be found in the SM and Table S1. The concentration of dyes in the fluorescence imaging experiments was 3 × 10^−7^ M.

The effect of 2-(3-thienyl)ethanol, DCA or Thio-DCA compounds on cell viability was examined using the CellTiter Blue^TM^ viability assay (Promega Corp. Madison, WI, USA). Cells were plated in quadruplicate in black, cell culture-treated, 96-well, optical, flat-bottom plates at a density of 8 × 10^4^ cells/well. The viability results of different treatments were compared to those of the control (cells in the presence of deuterated DMSO) at both 99% or 95% confidence using the Holm-Bonferroni and Holm-Sidak tests and the nonparametric Kolmogorov-Smirnov and Mann-Whitney tests in Origin 9.0 (OriginLab Corp., Northampton, MA, USA).

Further details about all the experimental methods, procedures and instrumentation can be found in the SM.

### 4.3. Cell Culture

For the imaging and viability experiments in this work the cell lines 143B (CRL-8303), HeLa (CCL-2), MCF7 (HTB-22), MDA-MB-231 (CRM-HTB-26), and SKBR3 (HTB-30) were acquired from the American Type Culture Collection (ATCC, Manassas, VA, USA), and the lines MDA-MB-468 (ACC 738) and ZR751 (ACC 8701601) were obtained from the Leibniz-Institut DMSZ, German collection of microorganisms and cell cultures GmbH (DMSZ, Braunschweig, Germany). Further details about culture protocols and procedures can be found in the SM.

## Data Availability

The data presented in this study are openly available in University of Granada repository, Digibug at http://hdl.handle.net/10481/66449, reference number 10481/66449.

## Supplementary Materials

The following are available online at https://www.mdpi.com/1999-4923/13/2/254/s1: Scheme S1. Synthesis of compounds I and II from 1,3-propanediol.; Scheme S2. Bromination of 2-thiophenemethenol.; Scheme S3. Synthesis of compound 1 from 2-methoxyacridin-9(10H)-one.; Scheme S4. Synthetic route for the preparation of compound 2 starting from 4-bromo-3-methylphenol.; Scheme S5. Nucleophilic addition of 3-iodotiophene to ketone VIII to obtain xanthene 3.; Scheme S6. Synthesis of BODIPY derivative 4.; Figure S1. 1H-NMR (500 MHz) spectrum of compound II in CDCl3.; Figure S2. 13C-NMR (126 MHz) spectrum of compound II in CDCl3.; Figure S3. 1H-NMR (500 MHz) spectrum of compound 1 in MeOD.; Figure S4. 13C-NMR (126 MHz) spectrum of compound 1 in MeOD.; Figure S5. 1H-NMR (400 MHz) spectrum of compound V in CDCl3.; Figure S6. 13C-NMR (101 MHz) spectrum of compound V in CDCl3.; Figure S7. 1H-NMR (400 MHz) spectrum of compound VI in CDCl3.; Figure S8. 13C-NMR (101 MHz) spectrum of compound VI in CDCl3.; Figure S9. 1H-NMR (400 MHz) spectrum of compound VII in CDCl3.; Figure S10. 13C-NMR (101 MHz) spectrum of compound VII in CDCl3.; Figure S11. 1H-NMR (400 MHz) spectrum of compound 2 in MeOD.; Figure S12. 13C-NMR (101 MHz) spectrum of compound 2 in CDCl3.; Figure S13. 1H-NMR (500 MHz) spectrum of compound 3 in DMSO-d6.; Figure S14. 13C-NMR (126 MHz) spectrum of compound 3 in DMSO-d6.; Figure S15. 1H-NMR (400 MHz) spectrum of compound Thio-DCA in CDCl3.; Figure S16. 13C-NMR (75 MHz) spectrum of compound Thio-DCA in CDCl3.; Table S1. Dual-color FLIM instrumental settings for colocalization studies of each dye.; Figure S17. Absorption (**A**, **C**) and fluorescence emission (**B**, **D**) spectra of dyes 1 (**A**, **B**) and 4 (**C**, **D**) at different pH values. The excitation wavelength for the emission spectra was 375 nm for compound 1 and 495 nm for compound 4.; Figure S18. Absorption and fluorescence emission dependence with pH of dyes 2 (**A**–**C**) and 3 (**D**–**F**) in aqueous solution. **A** and **D**) Absorption spectra at different pH values of dyes 2 (**A**) and 3 (**D**). B and **E**) Global fittings of the A vs pH data to the general equilibrium equations [15,16] to obtain the ground state pKa values of dyes 2 (**B**) and 3 (**E**). (**C** and **F**) Fluorescence emission spectra (λex = 490 nm) of dyes 2 (**C**) and 3 (**F**) in aqueous solution at different pH values.; Figure S19. Average fluorescence lifetime, τ, of dye 3 in methanol:glycerine mixtures of different viscosity at 20, 30 and 40 °C.; Figure S20. Photostability of dyes 1–4 during 2 h of continuous irradiation.; Figure S21. Representative dual-color FLIM images of compound 1 in 143B cells and ρ0206 cells after 20 min of incubation with MT. Panels (**A**) and (**B**) show FLIM images on a pseudo-color scale (between 0 and 17 ns) of the dye’s detection channel (left) and the MT detection channel (right). These examples were performed on human osteosarcoma 143B cells (**A**) and ρ0206 cells (**B**). The latter are tumor cells depleted of mitochondrial DNA, thus displaying an extreme metabolic phe-notype due to the absence of respiration [10]. Panels (**C**) and (**D**) show the colocalization images of 1 (green) and MT (red) in 143B cells (**C**) and ρ0206 cells (**D**). Scale bars represent 10 μm. Panels (**E**) and (**F**) show intensity traces in both channels for the depicted lines in images in panels C (for 143B cells) and D (for ρ0206 cells), respectively.; Figure S22. Representative dual-color, super-resolution optical fluctuation imaging (SOFI) of 1 (green channel) and MT (red channel) in formaldehyde-fixed HeLa cells, and intensity plots of the profile lines. Scale bars represent 5 μm.; Figure S23. Mitochondrial localization of compound 1 in 143B cells, after 20 min of incubation with MT, from dual-color FLIM images. Left panels represent the raw intensity images in the 1 channel and the MT channel. Central panels represent the selected region of interest, in cyan for compound 1 and red for MT. Rightmost panels are the overlaid images, with colocalized pixels represented in white color. Scale bars represent 10 μm.; Figure S24. FLIM imaging of compound 1 in 143B cells. The images show the fluorescence lifetime of 1 depicted on a pseudocolor scale between 13 and 16 ns. The leftmost column of images shows colocalized pixels with mitochondria. The central column of images shows non colocalized pixels. The rightmost column of images shows the overall images. Scale bars represent 10 μm. The plots on the right panels represent the pixel distribution of fluorescence lifetime of 1 in each image, localized in mitochondria (black lines) or in other cellular subcompartments (red lines), and the overall lifetime distribution (green lines). Given that the acridone core has a lifetime that depends on the polarity of the microenvironment [18], these data clearly show that the mitochondria matrix is a less polar environment than cellular cytoplasm.; Figure S25. Representative dual-color FLIM images of compound 2 in 143B (**A**, **C**, and **E**) and ρ0206 cells (**B**, **D**, and **F**), after 20 min of incubation with MT. In the FLIM images (**A** and **B**), the dye’s detection channel (left) and the MT detection channel (right) are shown separately. The colocalization images (**C** and **D**) of the dye (green) and MT (red) are also shown. Intensity traces in both channels (**E** and **F**) are shown for the depicted lines (marked as 1, 2 and 3) in the colocalization panels. Scale bars represent 10 μm.; Figure S26. Mitochondrial localization of compound 2 in ρ0206 cells, after 20 min of incubation with MT, from dual-color FLIM images. Left panels represent the raw intensity images in the 2 channel and the MT channel. Central panels represent the selected region of interest, in cyan for compound 2 and red for MT. Rightmost panels are the overlaid images, with colocalized pixels represented in white color. Scale bars represent 10 μm.; Figure S27. Mitochondrial localization of compound 3 in MDA-MB-231 cells, after 20 min of incubation with MT, from dual-color FLIM images. Left panels represent the raw intensity images in the 3 channel and the MT channel. Central panels represent the selected region of interest, in cyan for compound 3 and red for MT. Rightmost panels are the overlaid images, with colocalized pixels represented in white color. Scale bars represent 10 μm.; Figure S28. FLIM imaging of compound 3 in MDA-MB-231 cells. The images show the fluorescence lifetime of 3 depicted on a pseudocolor scale between 3 and 6 ns. The leftmost column of images shows colocalized pixels with mitochondria. The central column of images shows non colocalized pixels. The rightmost column of images shows the overall images. Scale bars represent 10 μm. The plots on the right panels represent the pixel distribution of fluorescence lifetime of 3 in each image, localized in mitochondria (black lines) or in other cellular subcompartments (red lines), and the overall lifetime distribution (green lines).; Figure S29. Representative dual-color FLIM images of compound 4 in 143B (**A**, **C**, and **E**) and ρ0206 cells (**B**, **D**, and **F**), after 20 min of incubation with MT. In the FLIM images (**A** and **B**), the dye’s detection channel (left) and the MT detection channel (right) are shown separately. The colocali-zation images (**C** and **D**) of the dye (green) and MT (red) are also shown. Intensity traces in both channels (E and F) are shown for the depicted lines in the colocalization panels. Scale bars represent 10 μm.; Table S2. Pearson’s correlation coefficient (PCC) and Manders’ colocalization coefficient (MCC) values for the colocalization of dyes 1–4 with the mitochondria tracker MT.[a].; Figure S30. Representative dual-color fluorescence images of compound 1 (green) and the MT tracker (magenta) in 143B cells after 20 min of incubation with BAM15. Scatter plots represent the corre-lation of the intensity values in the green channel vs the red channel. Scale bars represent 10 μm.; Figure S31. Cell viability of SKBR3 breast cancer tumor cells treated with 2-(3-thienyl)ethanol at different doses. Error bars represent standard deviations. Line represents the fit to a dose-response function.
