# Supplementary material for: Chimeric Drug Design with a Noncharged Carrier for Mitochondrial Delivery
Source: Pharmaceutics. 2021 Feb 12;13(2):254. doi: 10.3390/pharmaceutics13020254 (PMC7918843; doi:10.3390/pharmaceutics13020254)
Supplement: Supplementary file 1 [file pharmaceutics-13-00254-s001.pdf]

pharmaceutics

MDPI

Supplementary Materials: Chimeric Drug Design with a NonCharged Carrier for Mitochondrial Delivery

Consuelo Ripoll, Pilar Herrero-Foncubierta, Virginia Puente-Muñoz, M. Carmen Gonzalez-Garcia, Delia Miguel, Sandra Resa, Jose M. Paredes, Maria J. Ruedas-Rama, Emilio Garcia-Fernandez, Mar Roldan, Susana Rocha, Herlinde De Keersmaecker, Johan Hofkens, Miguel Martin, Juan M. Cuerva and Angel Orte*

S1. Synthesis of the Compounds in this Work

S1.1. Synthesis of Precursor Reagents

S1.1.1. Synthesis of Compounds I and II

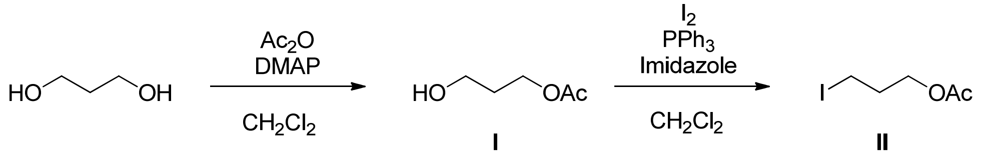

Scheme S1. Synthesis of compounds I and II from 1,3-propanediol.

__Compound I__. To a solution of 1,3-propanediol (6.00 g, 0.08 mol) in CH_2Cl_2 (30 mL), 4-dimethylaminopyrinide (DMAP) (10.51 g, 0.09 mol) was added and subsequently acetic anhydride (8 mL, 0.09 mol) was added dropwise. The mixture was stirred for 2 h at room temperature. Silica gel was then added and the solvent was removed. The crude was purified by flash chromatography (SiO_2, Hexane/EtOAc 6:4) to give compound **I** (4.01 g, 43%) as a colorless oil. ^1H and ^{13}C NMR spectra matched to those of the reported ones [1].

__Compound II.__ To a solution of I_2 (12.92 g, 0.05 mol) in anhydrous CH_2Cl_2 (50 mL), triphenylphosphine (12.92 g, 0.05 mol) was added thus giving a brown-yellow solution. Then, imidazole (7.63 g, 0.11 mol) was added, changing the color to light yellow. Next, compound **I** (4.01 g, 0.03 mol) was added and the mixture was stirred at room temperature until consumption of the starting material (checked by TLC, around 1–2 h). Silica gel was then added and the solvent was removed. The crude was purified by flash chromatography (SiO_2, Hexane/EtOAc 9:1) to give the compound **II** (6.43 g, 84%) as a yellow oil. ^1H NMR (500 MHz, $CDCl_3$) δ 4.13 (t, J = 6.1 Hz, 2H), 3.22 (t, J = 6.8 Hz, 2H), 2.14 (quint, J = 6.6 Hz, 2H), 2.06 (s, 3H). ^{13}C NMR (126 MHz, $CDCl_3$) δ 171.0 (C), 64.2 (CH_2), 32.5 (CH_2), 21.0 (CH_3), 1.5 (CH_2). HRMS (ESI): m/z $[M + Na]^+$ calcd for $C_5H_9O_2INa$: 250.9539; found: 250.9535.

S1.1.2 Synthesis of Compound III

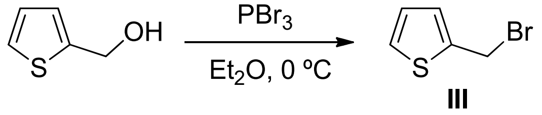

Scheme S2. Bromination of 2-thiophenemethenol.

To a solution of 2-thiophenemethenol (200 mg, 1.75 mmol) in anhydrous Et_2O at 0 °C, PBr_3 (0.1 mL, 0.88 mmol) was added dropwise. The mixture was stirred for 30–45 minutes at room temperature and then quenched by addition of methanol, diluted with water and extracted with Et_2O. The organic layer was separated and dried with anhydrous Na_2SO_4 and the solvent was removed to give the compound **III** as a yellow liquid in quantitative

yield. This compound was used in the next step without further purification. ^{1}H and ^{13}C NMR spectra matched to the reported ones [2].

S1.1.3 Synthesis of Compound IV

Precursor **IV** was synthesized by the addition of 2-methoxyacridin-9(10*H*)-one to previously synthesized iodide **II** following a previously described procedure [3]. ^{1}H and ^{13}C NMR spectra matched to those of the reported one [4].

S1.2. Synthesis of Compound 1

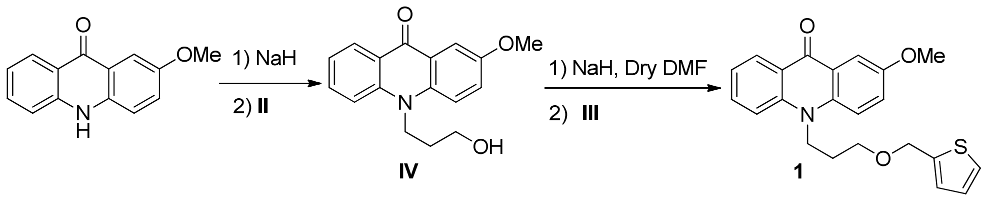

Scheme S3. Synthesis of compound 1 from 2-methoxyacridin-9(10H)-one.

To a solution of **IV** (68 mg, 0.24 mmol) in anhydrous dimethylformamide, DMF (5 mL), NaH (60% in mineral oil, 30 mg, 0.72 mmol) was added. The resulting mixture was stirred for 10–15 minutes and then compound **III** (51 mg, 0.29 mmol) was added. The reaction mixture was stirred for another 24 h. After this time, the reaction was quenched by dropwise addition of water. The mixture was diluted with EtOAc and washed with HCl 10% (×3), the organic layer was separated and dried with anhydrous Na_2SO_4 and the solvent was removed. The residue was purified by flash chromatography (SiO_2, Hexane/EtOAc 7:3) to give compound **1** (51 mg, 75%) as a yellow oil. An additional wash with hexane/dichloromethane mixtures gave compound **1** as yellow solid. ^{1}H NMR (500 MHz, MeOD) δ 8.41 (dd, *J* = 8.1, 1.6 Hz, 1H), 7.80 (d, *J* = 3.2 Hz, 1H), 7.78–7.71 (m, 3H), 7.41 (dd, *J* = 5.1, 1.2 Hz, 1H), 7.37 (dd, *J* = 9.4, 3.1 Hz, 1H), 7.28 (ddd, *J* = 7.9, 6.4, 1.3 Hz, 1H), 7.06 (dd, *J* = 3.4, 1.1 Hz, 1H), 7.01 (dd, *J* = 5.1, 3.5 Hz, 1H), 4.72 (s, 2H), 4.57–4.51 (m, 2H), 3.89 (s, 3H), 3.63 (t, *J* = 5.5 Hz, 2H), 2.15–2.07 (m, 2H). ^{13}C NMR (126 MHz, MeOD) δ 179.1 (C), 156.1 (C), 142.7 (C), 142.4 (C), 138.1 (C), 135.3 (CH), 128.1 (CH), 127.7 (CH), 127.5 (CH), 126.9 (CH), 126.2 (CH), 123.7 (C), 122.34 (CH), 122.28 (C), 118.6 (CH), 116.6 (CH), 106.9 (CH), 68.6 (CH_2), 67.9 (CH_2), 56.1 (CH_3), 44.4 (CH_2), 29.1 (CH_2). HRMS (ESI): *m*/*z* $[M + Na]^+$ calcd for $C_{22}H_{21}NNaO_3S$: 402.1140 found: 402.1123.

S1.3. Synthesis of Compound 2

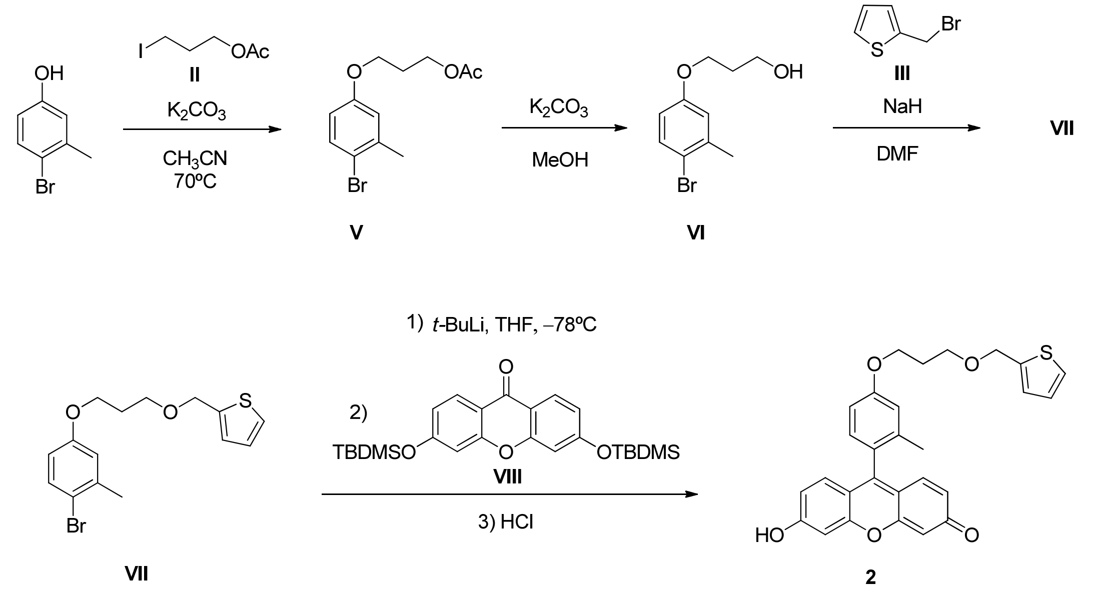

Scheme S4. Synthetic route for the preparation of compound 2 starting from 4-bromo-3-methylphenol.

<u>Compound V</u>. To a solution of commercially available 4-bromo-3-methylphenol (300 mg, 1.60 mmol) in CH_3CN (10 mL) at 70 °C, K_2CO_3 (266 mg, 1.93 mmol) and compound **II** (439 mg, 1.93 mmol) were added. The reaction mixture was stirred at 70 °C for 24 h. Next, K_2CO_3 was filtrated, silica gel was added to the mixture and the solvent was removed. The residue was purified by flash chromatography (SiO_2, Hexane/EtOAc 9:1) to give compound **V** (325 mg, 71%) as a light yellow liquid. ^1H NMR (400 MHz, $CDCl_3$) δ 7.39 (d, *J* = 8.7 Hz, 1H), 6.78 (d, *J* = 3.0 Hz, 1H), 6.60 (dd, *J* = 8.7, 3.0 Hz, 1H), 4.25 (t, *J* = 6.3 Hz, 2H), 4.00 (t, *J* = 6.1 Hz, 2H), 2.36 (s, 3H), 2.10 (quint, *J* = 6.3 Hz, 2H), 2.05 (s, 3H). ^{13}C NMR (101 MHz, $CDCl_3$) δ 171.2 (C), 158.1 (C), 139.0 (C), 133.0 (CH), 117.3 (CH), 115.7 (C), 113.6 (CH), 64.7 (CH_2), 61.3 (CH_2), 28.7 (CH_2), 23.3 (CH_3), 21.1 (CH_3). HRMS (ESI): *m*/*z* $[M + Na]^+$ calcd for $C_{12}H_{15}O_3BrNa$: 309.0096 found: 309.0092.

<u>Compound VI</u>. To a solution of compound **V** (325 mg, 1.13 mmol) in MeOH (10 mL), K_2CO_3 (469 mg, 3.40 mmol) was added. The mixture was stirred at room temperature for 15 min. Silica gel was then added and the solvent was removed. The crude was purified by flash chromatography (SiO_2, Hexane/EtOAc 6:4) to give the compound **VI** (234 mg, 84%) as a light yellow liquid. ^1H NMR (400 MHz, $CDCl_3$) δ 7.39 (d, *J* = 8.7 Hz, 1H), 6.80 (d, *J* = 3.0 Hz, 1H), 6.62 (dd, *J* = 8.7, 3.0 Hz, 1H), 4.08 (t, *J* = 6.0 Hz, 2H), 3.85 (q, *J* = 5.5 Hz, 2H), 2.36 (s, 3H), 2.03 (quint, *J* = 6.0 Hz, 2H), 1.69 (t, *J* = 4.7 Hz, 1H). ^{13}C NMR (101 MHz, $CDCl_3$) δ 158.1 (C), 139.0 (C), 133.0 (CH), 117.3 (CH), 115.7 (C), 113.6 (CH), 66.0 (CH_2), 60.5 (CH_2), 32.1 (CH_2), 23.3 (CH_3). HRMS (ESI): *m*/*z* $[M + Na]^+$ calcd for $C_{10}H_{13}O_2BrNa$: 266.9991 found: 266.9980.

<u>Compound VII</u>. To a solution of compound **VI** (234 mg, 0.95 mmol) in anhydrous DMF (6 mL), NaH (60% in mineral oil, 115 mg, 2.87 mmol) and compound **III** (254 mg, 1.43 mmol) were added. The mixture was stirred at room temperature for 16 h. After this time, the mixture was quenched by dropwise addition of water, diluted with EtOAc and washed with HCl 10%. The organic layer was dried with anhydrous Na_2SO_4 and the solvent was removed. The residue was purified by flash chromatography (SiO_2, Hexane/EtOAc 98:2) to give compound **VII** (279 mg, 86%) as a light yellow liquid. ^1H NMR (400 MHz, $CDCl_3$) δ 7.38 (d, *J* = 8.7 Hz, 1H), 7.27 (dd, *J* = 4.9, 1.4 Hz, 1H), 7.00–6.98 (m, 1H), 6.96 (dd, *J* = 4.9, 3.5 Hz, 1H), 6.78 (d, *J* = 3.0 Hz, 1H), 6.60 (dd, *J* = 8.7, 3.0 Hz, 1H), 4.68 (s, 2H), 4.03 (t, *J* = 6.3 Hz, 2H), 3.65 (t, *J* = 6.1 Hz, 2H), 2.36 (s, 3H), 2.05 (quint, *J* = 6.2

Hz, 2H). ^{13}C NMR (101 MHz, $CDCl_3$) δ 158.4 (C), 141.3 (C), 138.8 (C), 132.9 (CH), 126.8 (CH), 126.4 (CH), 125.9 (CH), 117.3 (CH), 115.5 (C), 113.7 (CH), 67.7 (CH_2), 66.5 (CH_2), 65.1 (CH_2), 29.8 (CH_2), 23.3 (CH_3). HRMS (ESI): *m/z* $[M+Na]^+$ calcd for $C_{15}H_{17}O_2SBrNa$: 363.0024 found: 363.0028.

Compound VIII. Compound **VIII** was prepared according to a previously described procedure. ^1H and ^{13}C NMR spectra matched to the reported ones [5].

Compound 2. To a solution of compound **VII** (279 mg, 0.82 mmol) in freshly distilled THF (4 mL) under an Ar atmosphere at –78 °C, *t*-BuLi (1.7 M in hexane, 0.96 mL, 1.64 mmol) was added dropwise. After keeping the reaction at that temperature for 20 minutes, a solution of compound **VIII** (187 mg, 0.41 mmol) in THF (2 mL) was slowly added. Then, the mixture was stirred at –78 °C for 15 min and then allowed to reach room temperature. The reaction was monitorized by TLC. After consumption of compound **VIII**, HCl 10% (1 mL) was added promoting a color change from light yellow to orange. Finally, solvent was removed and residue was purified by flash chromatography (SiO_2, CH_2Cl_2/MeOH 9:1) to give compound **2** (130 mg, 66%) as an orange solid. ^1H NMR (400 MHz, MeOD) δ 7.36 (dd, *J* = 5.1, 1.2 Hz, 1H), 7.12 (d, *J* = 8.4 Hz, 1H), 7.08 (d, *J* = 9.7 Hz, 2H), 7.05–7.03 (m, 1H), 7.00 (d, *J* = 2.5 Hz, 1H), 6.99–6.94 (m, 2H), 6.68–6.64 (m, 4H), 4.72 (s, 2H), 4.17 (t, *J* = 6.2 Hz, 2H), 3.72 (t, *J* = 6.1 Hz, 2H), 2.09 (quint, *J* = 6.2 Hz, 2H), 2.02 (s, 3H). ^{13}C NMR (101 MHz, MeOD) δ 179.0 (C), 161.5 (C), 159.7 (C), 156.5 (C), 142.5 (C), 138.9 (C), 132.3 (CH), 131.5 (CH), 127.59 (CH) 127.58 (CH), 126.8 (CH), 126.0 (C), 123.4 (CH), 117.6 (CH), 115.5 (C), 113.3 (CH), 104.5 (CH), 68.3 (CH_2), 67.4 (CH_2), 66.0 (CH_2), 30.7 (CH_2), 20.0 (CH_3). HRMS (ESI): *m/z* $[M + H]^+$ calcd for $C_{28}H_{25}O_5S$: 473.1417 found: 473.1416.

S1.4. Synthesis of Compound 3

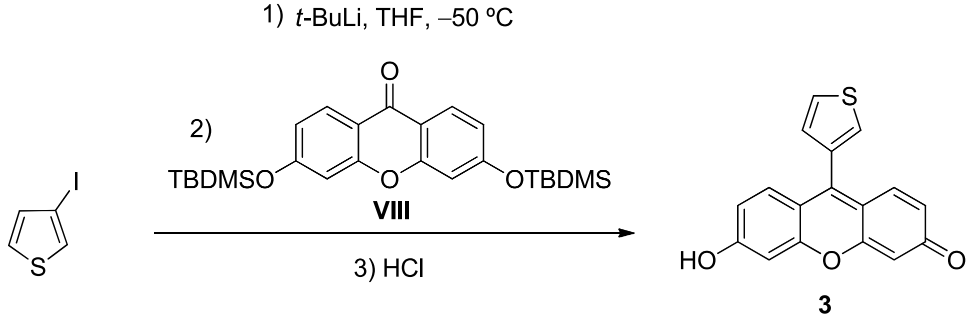

Scheme S5. Nucleophilic addition of 3-iodotiophene to ketone VIII to obtain xanthene 3.

To a solution of 3-iodothiophene (118 mg, 0.56 mmol) in freshly distilled THF (2 mL) under Ar atmosphere at –50 °C, *t*-BuLi (1.7 M in hexane, 0.66 mL, 1.12 mmol) was added dropwise. After keeping the reaction at that temperature for 20 minutes, a solution of compound **VIII** (128 mg, 0.28 mmol) in THF (2 mL) was slowly added. Then, the mixture was stirred at –50 °C for 15 min and then allowed to reach room temperature. The reaction was monitored by TLC. After consumption of compound **VIII**, HCl 10% (1 mL) was added promoting a color change from light yellow to orange. Finally, solvent was removed and residue was purified by flash chromatography (SiO_2, CH_2Cl_2/MeOH 8:2) to give the compound **3** (48 mg, 59%) as an orange solid. ^1H NMR (500 MHz, DMSO-d_6) δ 7.93–7.87 (m, 2H), 7.32 (dd, *J* = 4.7, 1.4 Hz, 1H), 7.22 (d, *J* = 9.3 Hz, 2H), 6.64 (bs, 4H). ^{13}C NMR (126 MHz, DMSO-d_6) δ 145.4 (C), 132.2 (C), 130.5 (CH), 129.3 (CH), 128.0 (CH), 127.7 (CH). Several carbons are not observed. HRMS (EI): *m/z* $[M]^+$ calcd for $C_{17}H_{10}O_3S$: 294.0351 found: 294.0339.

S1.5. Synthesis of Compound 4

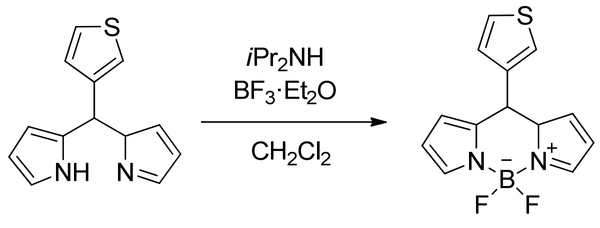

Scheme S6. Synthesis of BODIPY derivative 4.

First, precursor **IX** was prepared according to a previously described procedure. ^1H and ^{13}C NMR spectra matched those of the reported ones [6].

Then, compound **4** was prepared as follows. To a solution of compound **IX** (100 mg, 0.44 mmol) in CH_2Cl_2 (5 mL), diisopropylamine (0.3 ml, 2.12 mmol) was added. The mixture was stirred for 1 h at room temperature. Then $BF_3·OEt_2$ (0.55 ml, 2.12 mmol) was added and the resulting mixture was heated under reflux. The reaction was monitored by TLC until consumption of starting materials. The mixture was allowed to cool to room temperature, diluted with water and washed with CH_2Cl_2, the organic layer was separated and dried with anhydrous Na_2SO_4 and the solvent was removed. The residue was purified by flash chromatography (SiO_2, CH_2Cl_2/MeOH 9:1) to give the compound **4** (65 mg, 53%) as a yellow-brown solid. ^1H and ^{13}C NMR spectra matched those of the reported ones [6].

S2. ^{1}H-NMR and ^{13}C-NMR Spectra of New Compounds

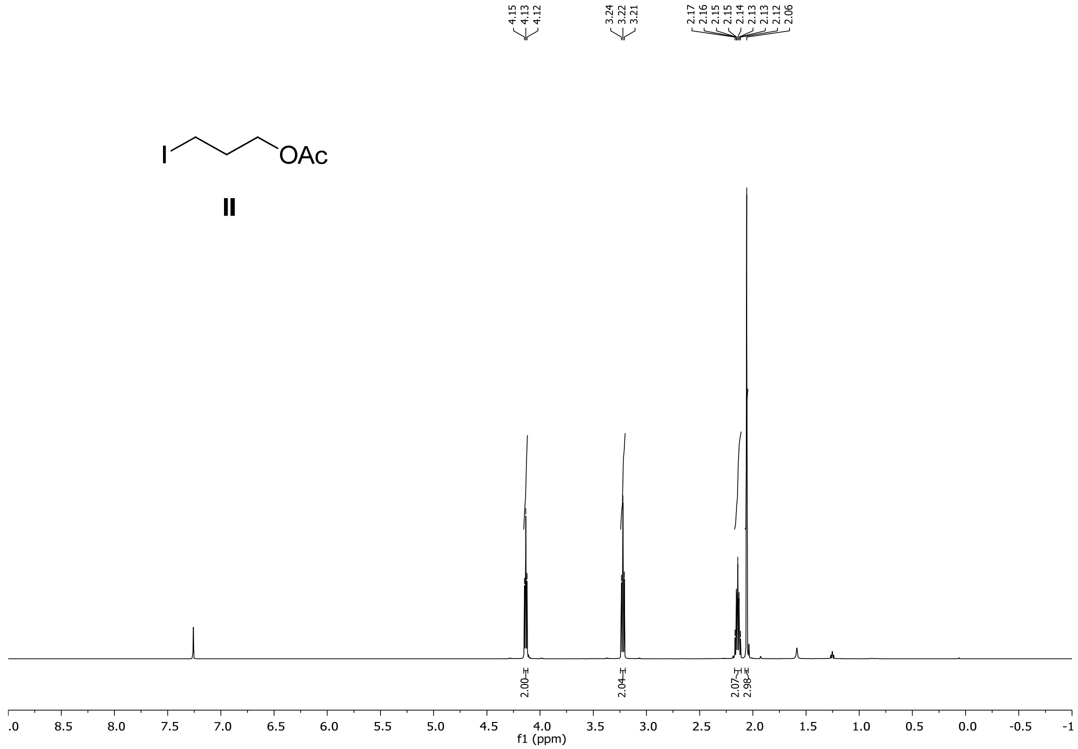

Figure S1. ^{1}H-NMR (500 MHz) spectrum of compound II in $CDCl_3$.

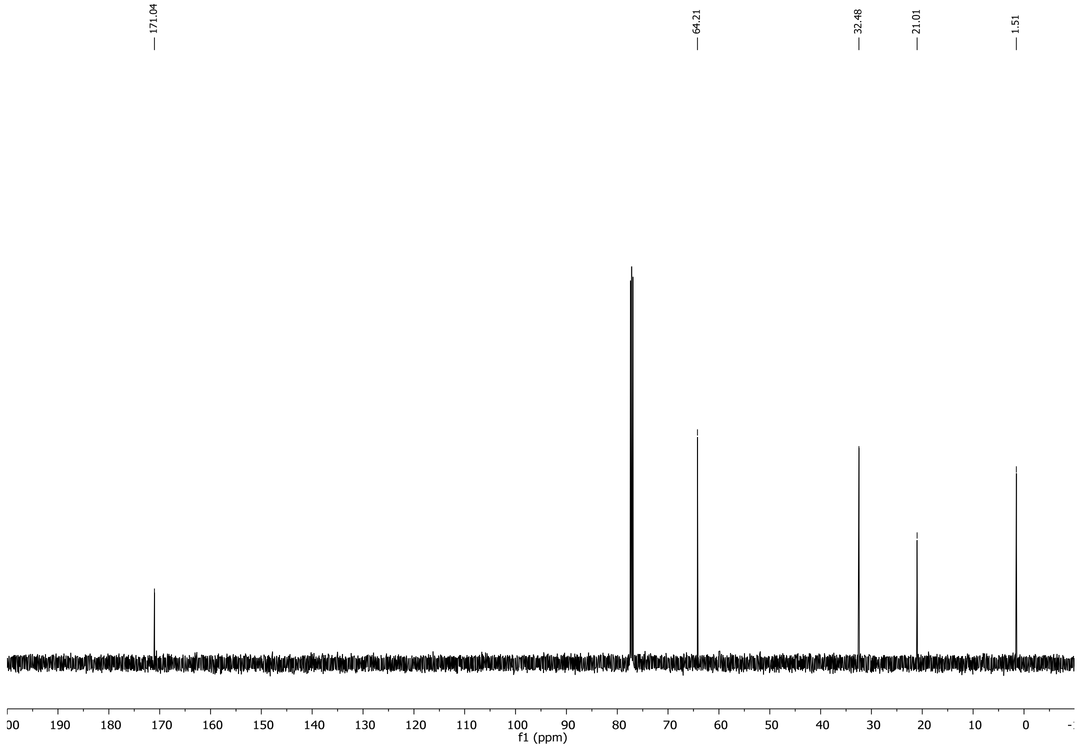

Figure S2. ^{13}C-NMR (126 MHz) spectrum of compound II in $CDCl_3$.

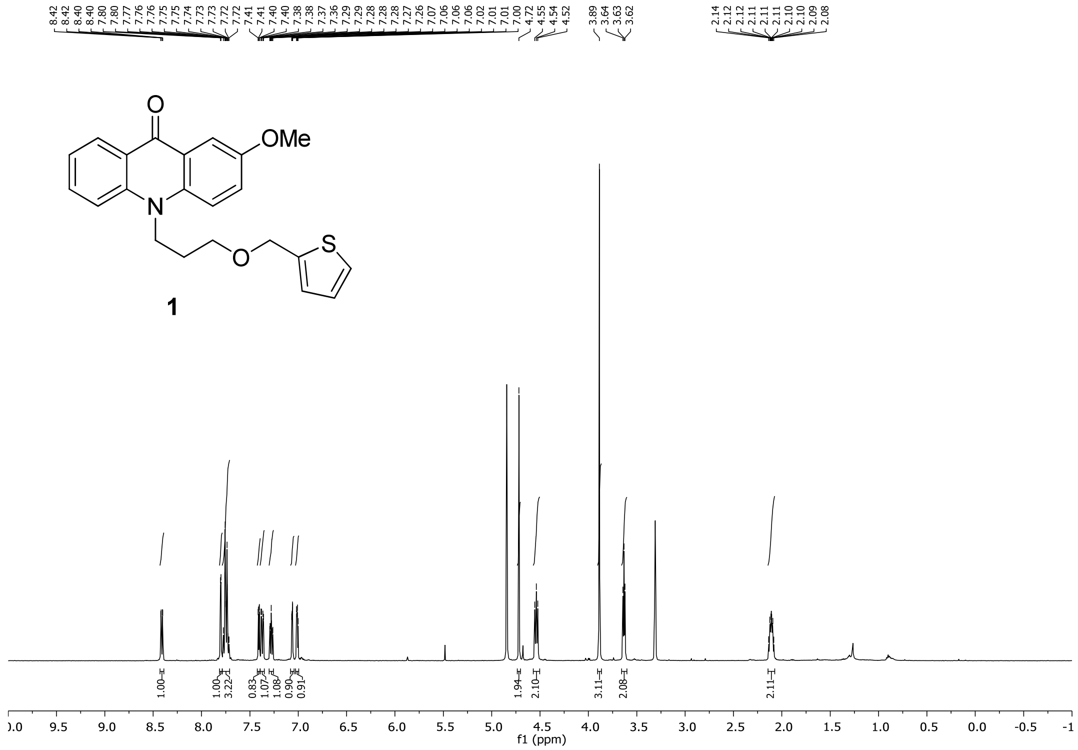

Figure S3. ^{1}H-NMR (500 MHz) spectrum of compound 1 in MeOD.

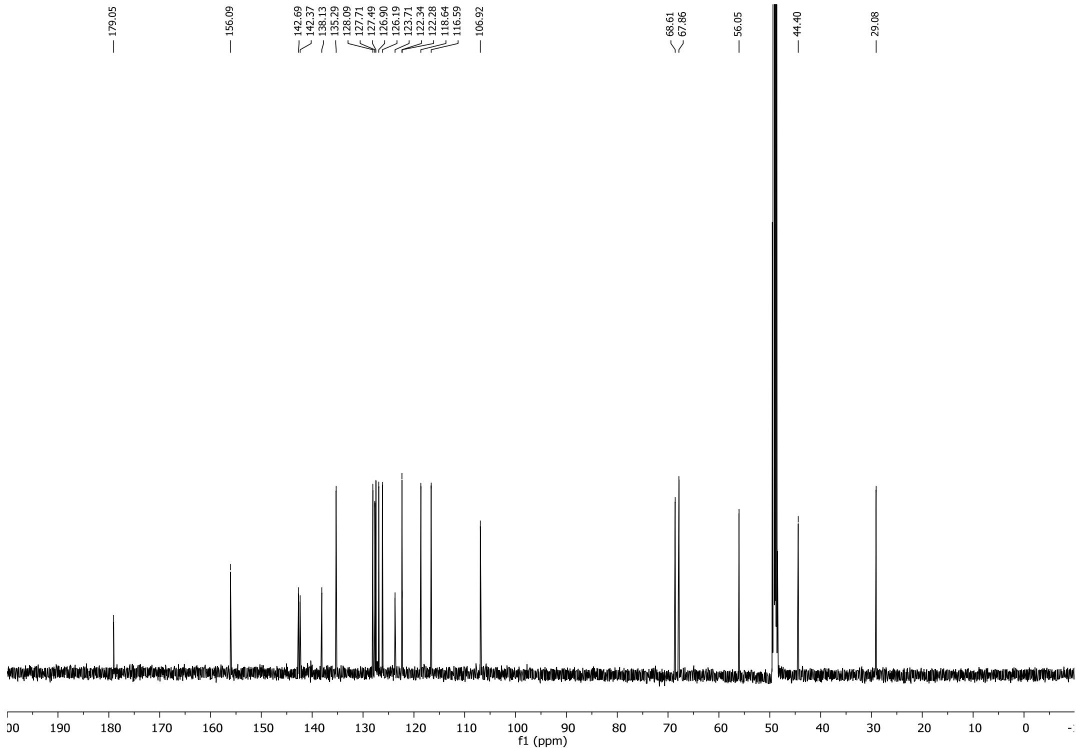

Figure S4. ^{13}C-NMR (126 MHz) spectrum of compound 1 in MeOD.

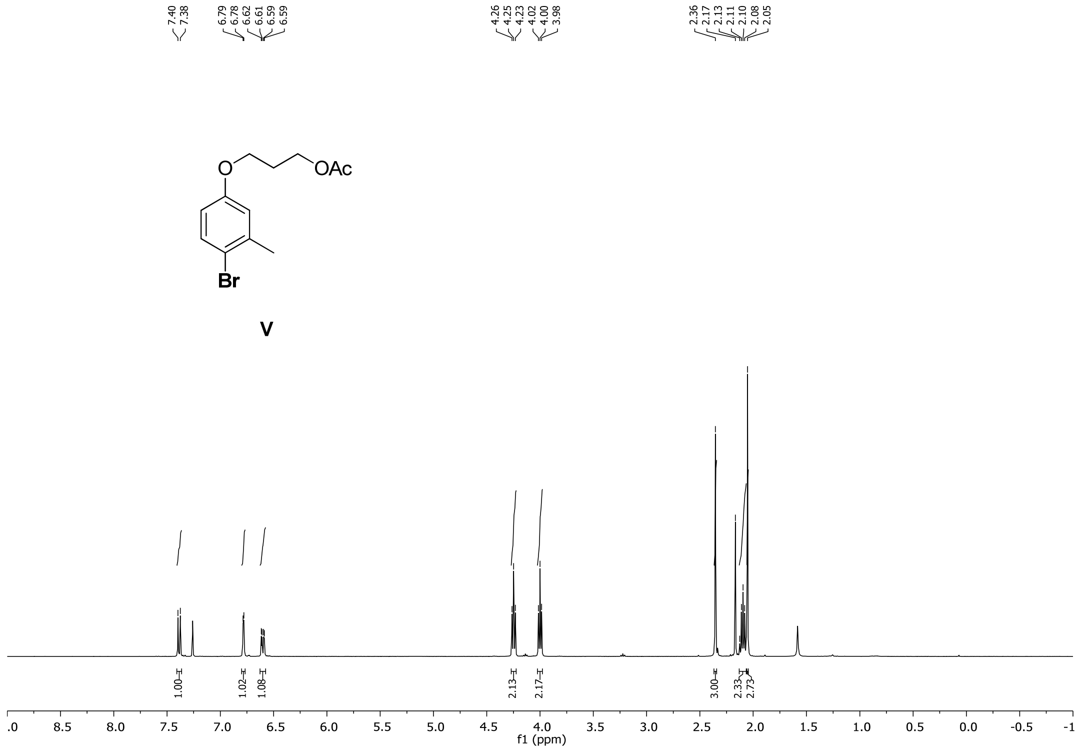

Figure S5. ^{1}H-NMR (400 MHz) spectrum of compound V in $CDCl_3$.

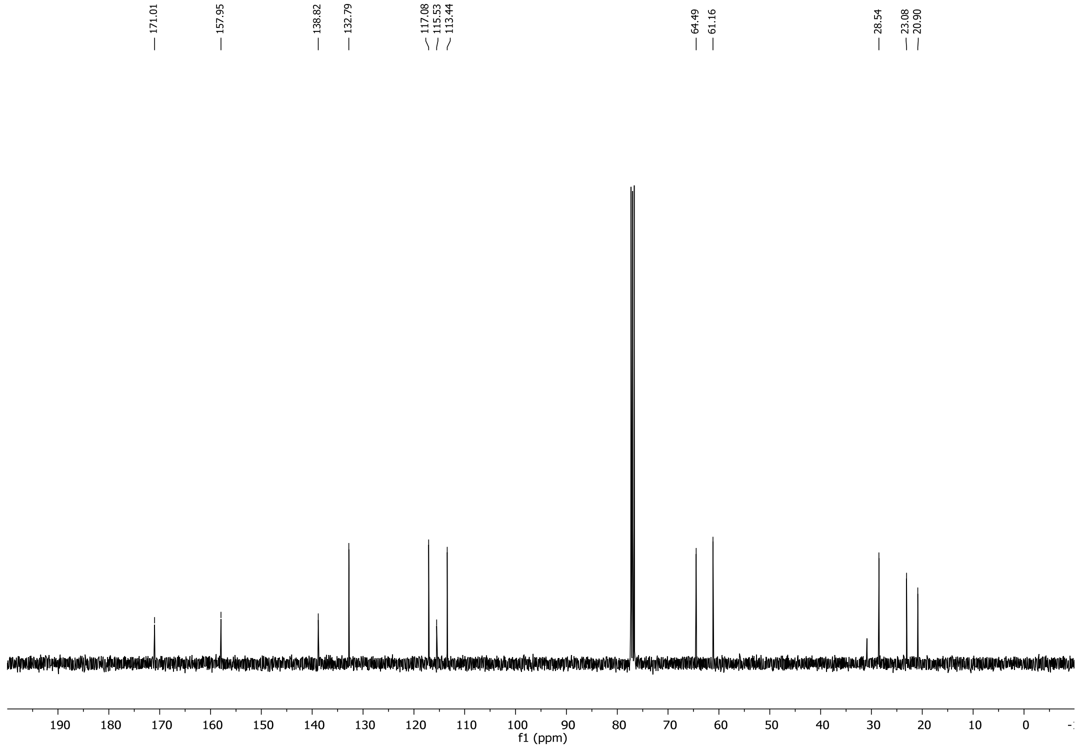

Figure S6. ^{13}C-NMR (101 MHz) spectrum of compound V in $CDCl_3$.

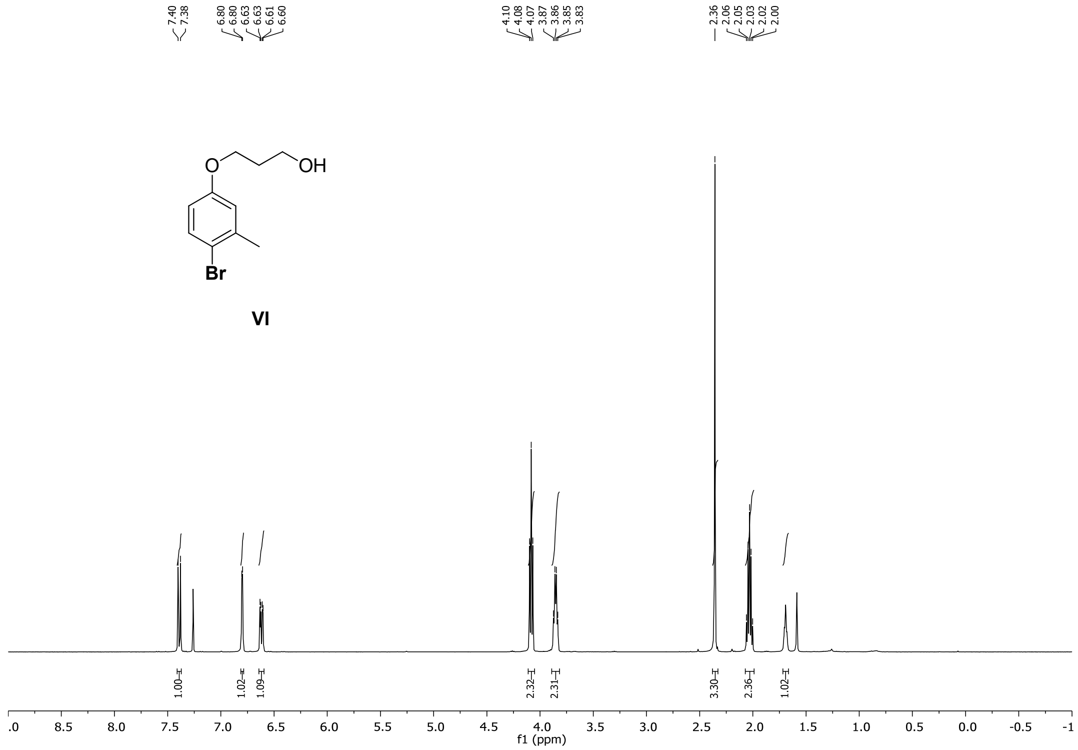

Figure S7. ^{1}H-NMR (400 MHz) spectrum of compound VI in $CDCl_3$.

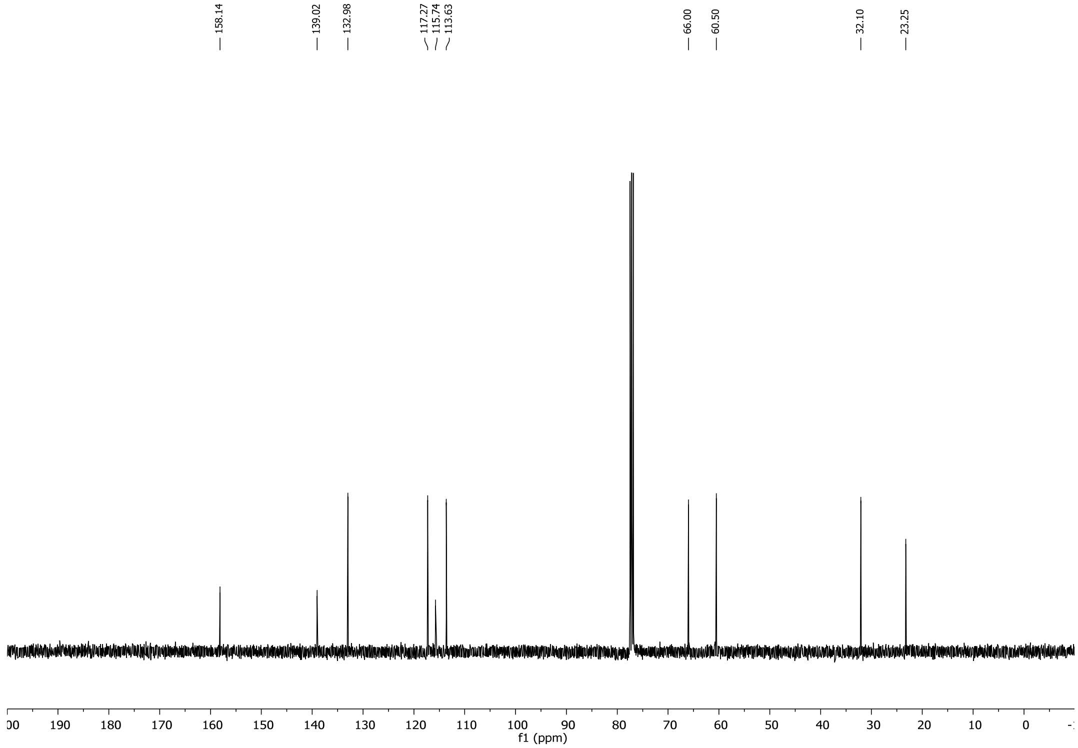

Figure S8. ^{13}C-NMR (101 MHz) spectrum of compound VI in $CDCl_3$.

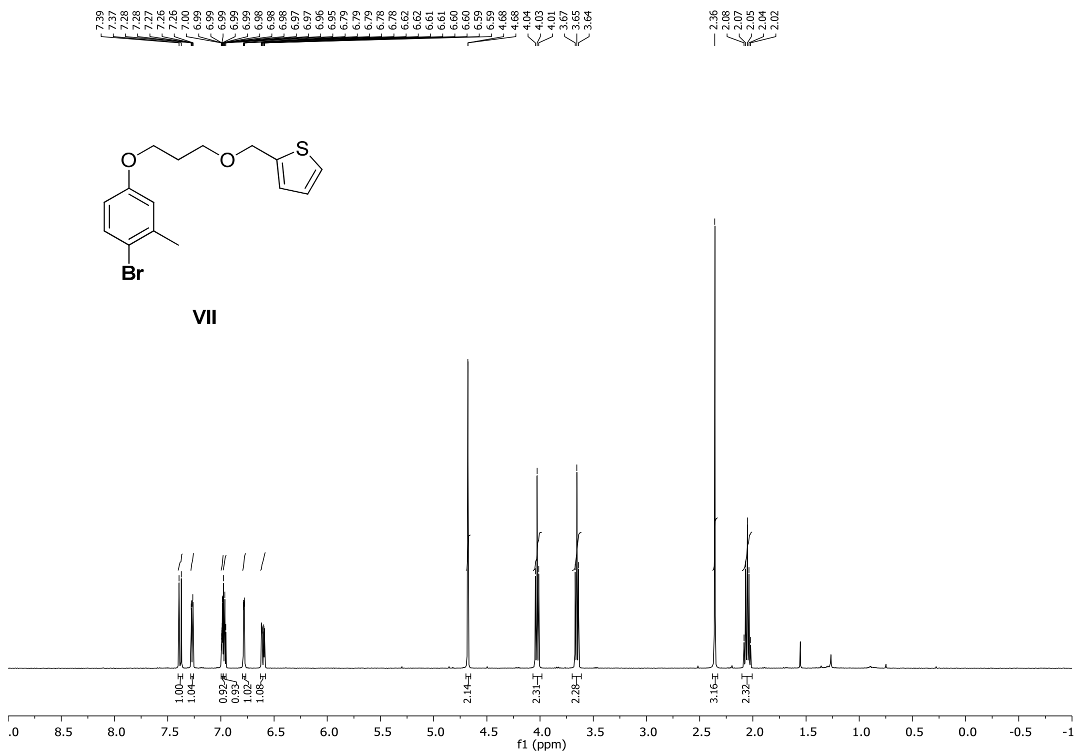

Figure S9. ^{1}H-NMR (400 MHz) spectrum of compound VII in $CDCl_3$.

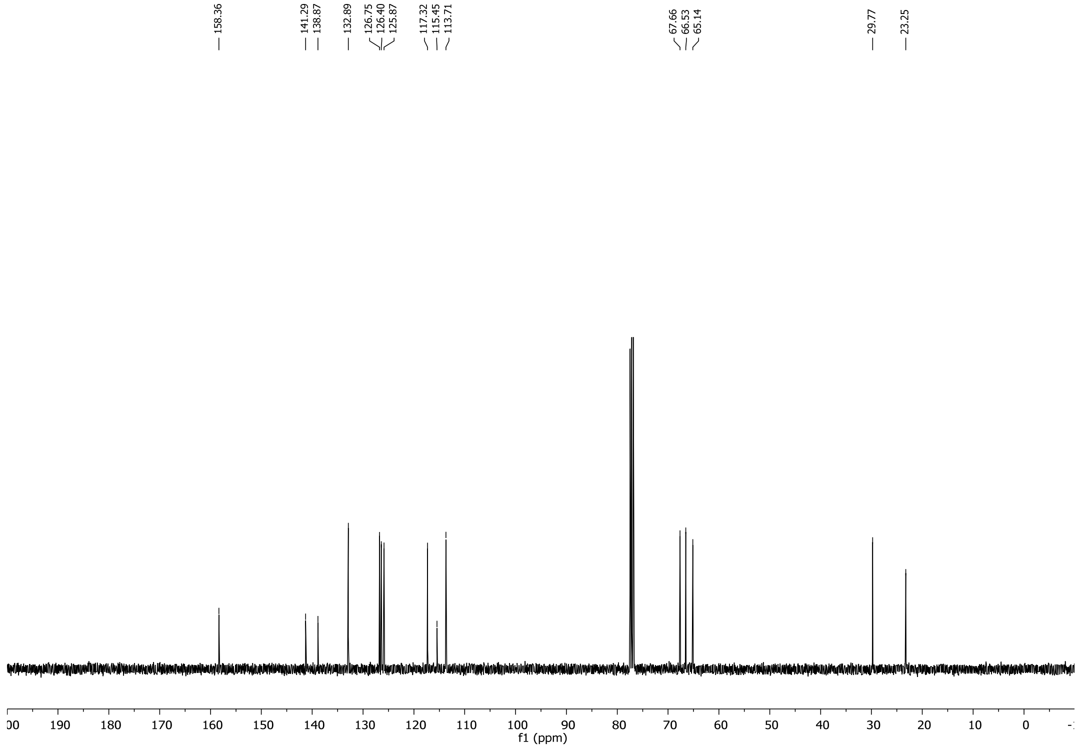

Figure S10. ^{13}C-NMR (101 MHz) spectrum of compound VII in $CDCl_3$.

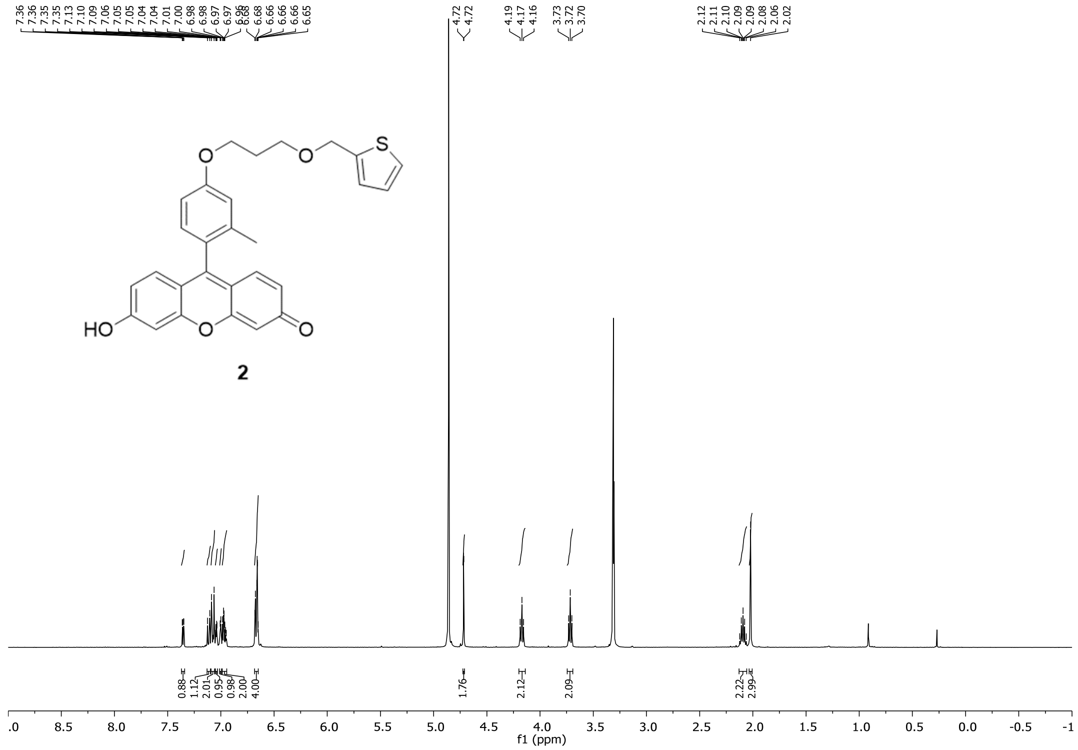

Figure S11. ^{1}H-NMR (400 MHz) spectrum of compound 2 in MeOD.

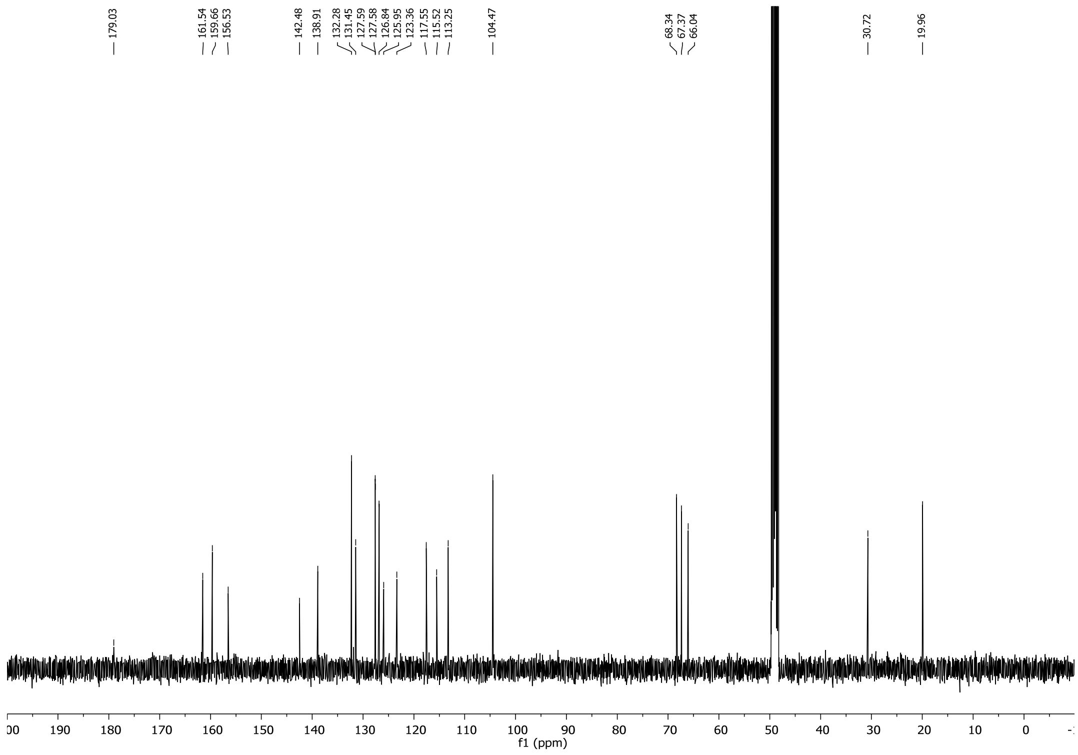

Figure S12. ^{13}C-NMR (101 MHz) spectrum of compound 2 in $CDCl_3$.

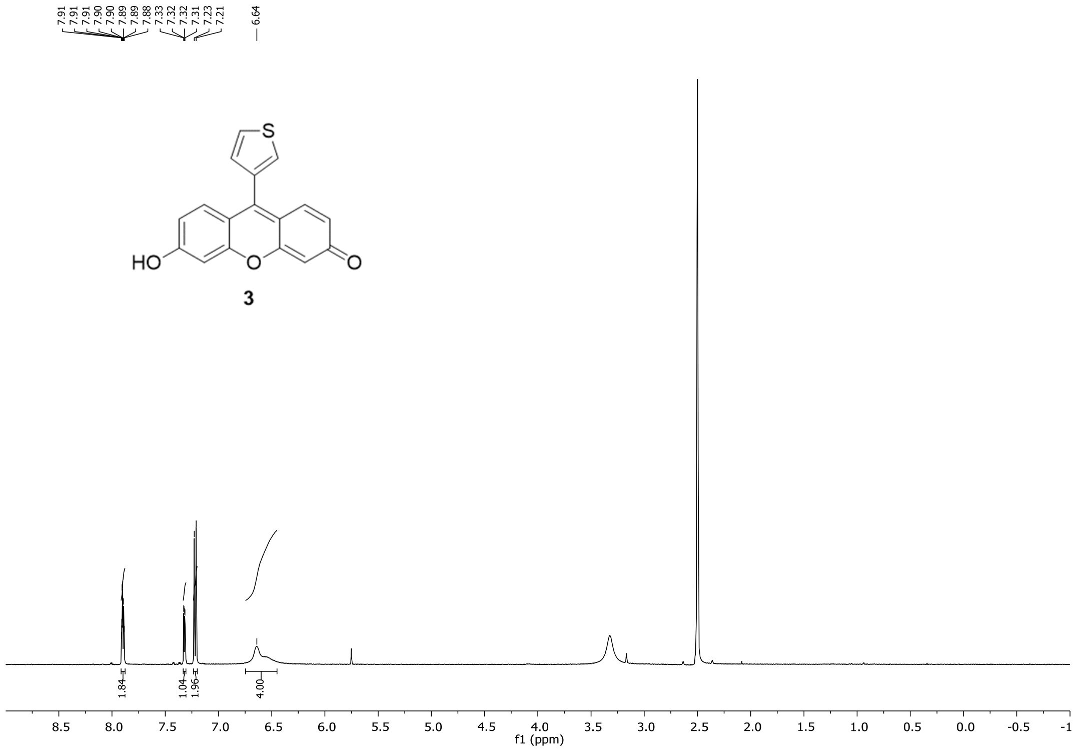

Figure S13. ^{1}H-NMR (500 MHz) spectrum of compound 3 in DMSO-d_6.

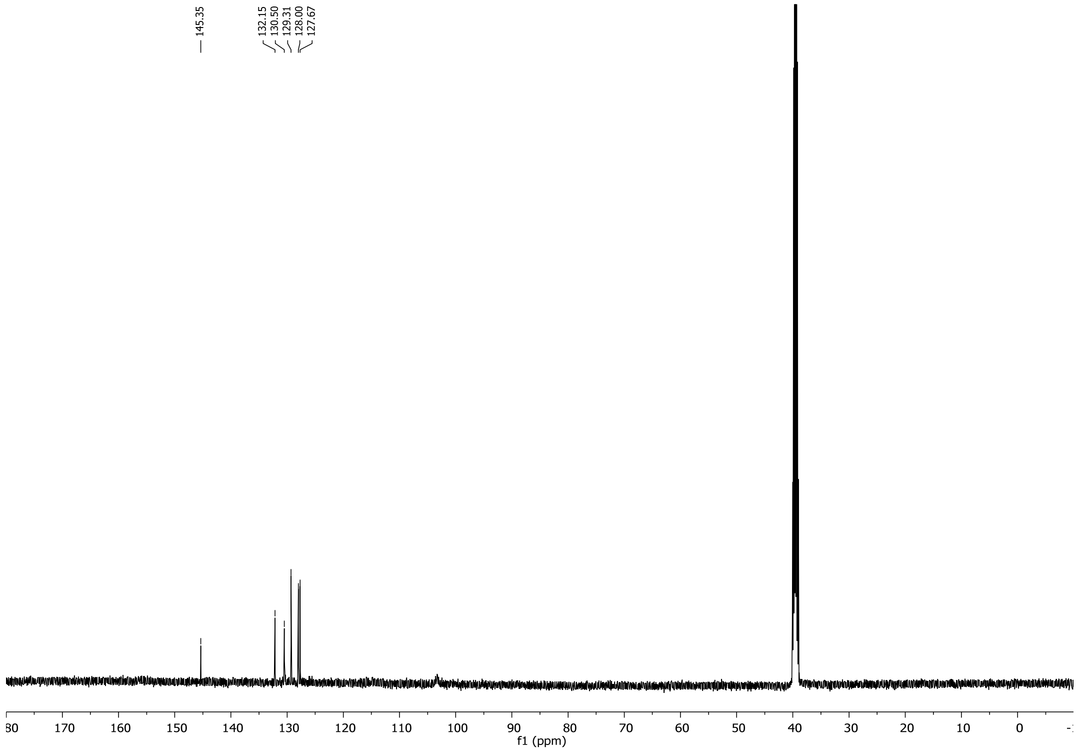

Figure S14. ^{13}C-NMR (126 MHz) spectrum of compound 3 in DMSO-d_6.

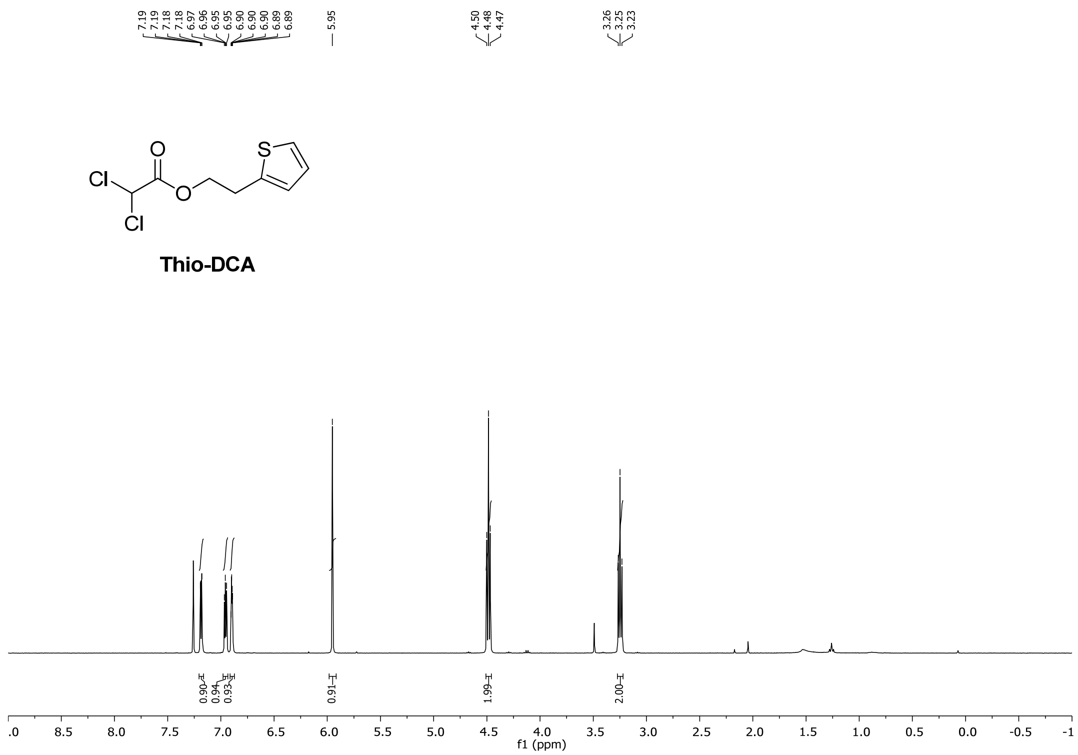

Figure S15. ^{1}H-NMR (400 MHz) spectrum of compound Thio-DCA in $CDCl_3$.

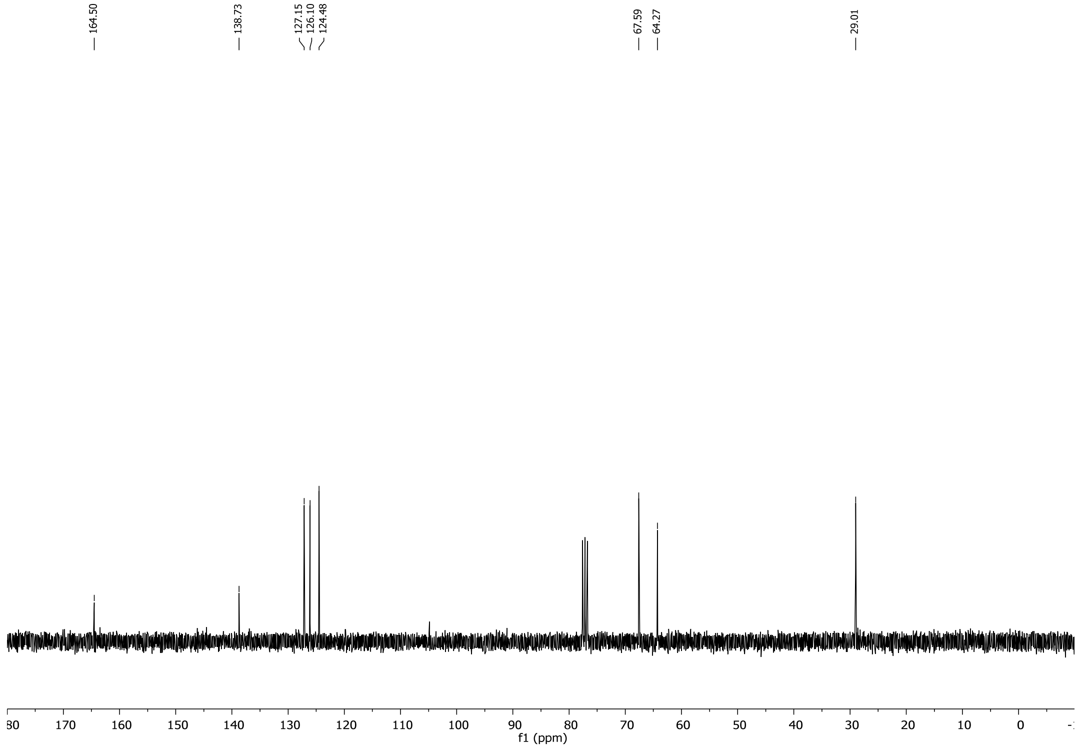

Figure S16. ^{13}C-NMR (75 MHz) spectrum of compound Thio-DCA in $CDCl_3$.

S3. Supplementary Experimental Procedures

S3.1. Spectroscopy Measurements

Absorption spectra of the different dyes in aqueous solutions were obtained on a Lambda 650 UV–visible spectrophotometer (PerkinElemer, Whaltam, MA, USA). The steady-state fluorescence emission and excitation spectra were collected using a Jasco FP-8300 spectrofluorometer (Jasco, Tokyo, Japan). Time-resolved fluorescence decay traces were obtained on a FluoTime 200 (PicoQuant, Berlin, Germany), equipped with 375-nm, 440-nm, 470-nm, and 485-nm pulsed diode lasers as excitation sources, controlled by a PDL-800 laser driver (PicoQuant). Fluorescence decay traces were collected at three different emission wavelengths around the emission maximum of each dye, up to a total of 2×10^4 counts at the peak channel and using a 34 ps/channel time resolution. Global analysis was performed for an accurate determination of the fluorescence lifetimes.

Aqueous phosphate buffer (Sigma-Aldrich-Merck, Darmstadt, Germany) and Tris buffer (Sigma-Aldrich-Merck) were prepared by dilution of the commercial concentrate solution to the final 10 mM concentration, and the pH dropwise adjusted using 1 M solutions of HCl or NaOH. For the measurements of dye **3** in methanol:glycerine mixtures of different viscosity, the mixtures were prepared using different proportions ranging from 100% (*v*/*v*) methanol to 99% (*v*/*v*) glycerine. The density and viscosity of the mixtures were measured in quintuplicate using floating pycnometers and a Hoppler viscosimeter, respectively, at three different temperatures, on a temperature-controlled water bath.

S3.2. Dual-Color Confocal Fluorescence Microscopy

Multicolor confocal imaging of cells stained with MT and compound **2** or **3** was performed using a laser scanning microscope (Fluoview FV1000; Olympus, Tokyo, Japan). A 60× water immersion objective (UPLSAPO, NA = 1.20) was used for all the experiments. For imaging compound **2**, compound **3** or MT, the sample was excited with a 405 nm, 488 nm or 635 nm laser line, respectively. The excitation light was reflected using a DM405/488/559/635 dichroic (Olympus). The fluorescence from the synthesized compounds and MT was split using an SDM 510 or SDM 560 dichroic for compound **2** or **3**, respectively. Emission from compound **2** was collected between 410 and 490 nm, and emission from compound **3** was collected between 500 and 580 nm using a grating. For MT, a BA655-755 bandpass filter was used. To minimize the crosstalk between the channels, image acquisition was performed using consecutive excitation.

S3.3. Dual-Color Fluorescence Lifetime Imaging Microscopy (FLIM)

Colocalization studies using dual-color FLIM were performed on a MicroTime 200 fluorescence lifetime microscope system (PicoQuant). Different pulsed diode lasers were employed as excitation sources (all from the LDH series from PicoQuant), depending on the dye to be imaged (see Table S1). The laser heads were controlled with a Sepia II driver (PicoQuant) working at a 10 or 20 MHz repetition rate. When dual-color pulsed interleaved excitation (PIE) [7] was possible, for the alternating excitation of the studied dye and the MitoTracker Deep Red (MT) dye (Life Technologies, Carlsbad, CA, USA), the 635-nm pulsed laser (LDH-P-635, PicoQuant) was delayed to the middle of the detection window using a delay box (ORTEC DB463 delay box, Ametek, Berwyn, PA, USA). The excitation laser beams were directed into the specimen through an apochromatic oil immersion objective (100×, 1.4NA) of an inverted microscope system (IX-71, Olympus, Japan). The fluorescence light was collected back and filtered through the main dichroic and cut-off filters and spatially filtered through a 75-μm pinhole. Finally, the fluorescence was separated into two detection channels using a dichroic mirror or beam splitter; the first channel was dedicated to the tested fluorescent probe, whereas the second channel was dedicated to the red fluorescence from MT. Two single-photon avalanche diodes (SPCM-AQR 14, Perkin-Elmer Optoelectronics, Whaltam, MA, USA) were used as photon detection devices. Imaging reconstruction, photon counting, and data acquisition were realized using a TimeHarp 200 card (PicoQuant). The different areas were raster-scanned using a 512 × 512 pixel resolution, and a 60 ms/pixel acquisition time. FLIM analyses were performed by fitting the fluorescence decay traces obtained in the individual pixels, after

a 5 × 5 spatial binning, to a single exponential decay function using a reconstructed instrument response function and a parameter optimization based on the maximum likelihood estimator (MLE). FLIM imaging and colocalization image analyses were performed using home-coded scripts in SymPhoTime 32 (PicoQuant), MathCad 15.0 (PTC, Boston, MA, USA) and FiJi (distribution of ImageJ) [8,9].

Table S1. Dual-color FLIM instrumental settings for colocalization studies of each dye.

Compound[a]	Excitation Sources	Main Dichroic	Cut-off Filter	Detection Channel Splitter	Filter in Dye's Channel	Filter in MT's Channel
**1**	440 nm	440DCR	LP460	600DCXR	465/30	685/70
**2–4**	470 nm/635 nm in PIE[b]	470/635 dual-band	LP500	600DCXR	520/35	685/70

[a] See Chart 1 for structures. [b] PIE: Pulsed interleaved excitation.

Copyright: © 2021 by the authors. Licensee MDPI, Basel, Switzerland. This article is an open access article distributed under the terms and conditions of the Creative Commons Attribution (CC BY) license (http://creativecommons.org/licenses/by/4.0/).

S3.4. Cell Culture for Fluorescence Imaging

The human osteosarcoma cell line 143B (CRL-8303) was obtained from the American Type Culture Collection (ATCC, Manassas, VA, USA). The cells were cultured with high glucose Dulbecco's modified Eagle's medium (DMEM) containing GlutaMAX-I (4 mM), sodium pyruvate (1 mM) (Thermo Fisher Scientific, Waltham, MA, USA), 1% penicillin/streptomycin (Thermo Fisher Scientific), and 10% fetal bovine serum (FBS) (Thermo Fisher Scientific). The cells were subcultured every 3–4 days at a density of 2.4 or 5.6×10^3 cells/cm^2, and the media were refreshed the day before subculture.

$\varrho_0$206 cells were obtained from the parental osteosarcoma line 143B. These cells are depleted of mitochondrial DNA as a result of long-term passaging in the presence of low concentrations of ethidium bromide [10]. Accordingly, the genes for oxidative phosphorylation and mitochondrial respiration are knocked out of $\varrho_0$206 cells. The cells have mitochondrial organelles, but their metabolism is totally adapted for survival and to maintain their proliferative capacity. $\varrho_0$206 cells were kindly obtained from Giuseppe Attardi's laboratory. The cells were cultured in high-glucose DMEM containing GlutaMAX-I (4 mM), sodium pyruvate (1 mM) (Thermo Fisher Scientific), 2% penicillin/streptomycin (Thermo Fisher Scientific), and 10% FBS (Thermo Fisher Scientific). The cells also require an exogenous addition of 0.5% uridine (Sigma-Aldrich-Merck) and an extra concentration of sodium pyruvate (1 mM). The cells were subcultured every 3–4 days at a density of 5.6×10^3 or 1×10^4 cells/cm^2, and the media were refreshed the day before subculture.

The cell lines MCF7 (HTB-22), MDA-MB-231 (CRM-HTB-26), and SKBR3 (HTB-30) were acquired from ATCC, and the lines MDA-MB-468 (ACC 738) and ZR751 (ACC

8701601) were obtained from the Leibniz-Institut DMSZ, German collection of microorganisms and cell cultures GmbH (DMSZ, Braunschweig, Germany). MCF7 cell line was cultured with minimum essential medium (MEM, Thermo Fisher Scientific) with 10% FBS (Thermo Fisher Scientific), 1% GlutaMAX-I (Thermo Fisher Scientific), and 1% nonessential amino acids (NEAA). The cell line SKBR3 was cultured with modified McCoy's 5A medium, 1% glutamine (Thermo Fisher Scientific), 1% penicillin/streptomycin (Thermo Fisher Scientific), and 10% FBS (Thermo Fisher Scientific). The cell lines MDA-MB-231 and MDA-MB-468 were cultured in high-glucose DMEM containing GlutaMAX-I (4 mM), sodium pyruvate (1 mM) (Thermo Fisher Scientific), 1% penicillin/streptomycin (Thermo Fisher Scientific), and 10% FBS (Thermo Fisher Scientific). All these cell lines were subcultured every 3–4 days at a density of 1.35 or 1.1×10^6 cells/cm^2, and the media were refreshed the day before subculture. ZR751 cell line was cultured with a slightly different routine due to a longer doubling time than other lines. ZR751 cells were subcultured once a week, although the culture medium was changed twice between each seeding. RPMI-1640 medium (Thermo Fisher Scientific) was used for optimal growth, reconstituted with 10% FBS, 1% GlutaMAX-I, 1% sodium pyruvate and 1% penicillin/streptomycin. ZR751 cells were subcultured at a density of 1.8 $\times 10^6$ cells/cm^2 in T75 flasks.

HeLa cells (CCL-2, acquired from ATCC) were maintained in DMEM without phenol red (Thermo Fisher Scientific) and supplemented with 10% FBS (Thermo Fisher Scientific) at 37 °C under a humidified 5% CO_2 atmosphere. Before image acquisition the cells were washed 3 times with 1 mL of Hank's balanced salt solution (HBSS) (Thermo Fisher Scientific). The dye was added for 5 min and washed with HBSS. After that, 4% formaldehyde was added for cell fixation. After 30 min, the cells were washed with HBSS. Staining with the mitochondria tracker MT was performed according to the manufacturer's instructions. For imaging, cells were seeded in 35-mm, glass-bottom dishes (MatTeK, Ashland, MA, USA). Imaging was performed in HBSS at room temperature unless otherwise specified.

S3.5. Image Analysis

Image analysis and segmentation was performed as described previously [11,12], using home-scripted macros in FiJi [8]. In brief, background subtraction was performed by previously applying a 20 × 20 pixel median filter [13] and a subsequent manual threshold selection to select the regions of interest of both images (dye and MT).

The Pearson coefficient (r) was obtained through the next equation (Equation (S1)):

$$r = \frac{\sum(x-\bar{x})(y-\bar{y})}{\sqrt{\sum(x-\bar{x})^2 \sum(y-\bar{y})^2}} \tag{S1}$$

Where x and y are the pixel intensity values of the first and second images respectively and $\bar{x}$ and $\bar{y}$ are the mean of all the pixel matrix. We calculated r using a home-made macro in FiJi by obtaining the matrices $(x-\bar{x})$, $(y-\bar{y})$, $(x-\bar{x})^2$ and $(y-\bar{y})^2$ after applying the threshold.

Mander's colocalization coefficients were obtained to indicate the amount of mutually colocalized pixels for each channel using the JACoP plugin [14] in FiJi.

These treated images were stacked for visual inspection of colocalization, and obtain the segmented masks of coincident (assigned to mitochondria) or noncoincident pixels. These masks were subsequently applied to the dye's lifetime matrix to extract the lifetime distributions in each region.

S3.6. Cell Viability Assays

The DCA or Thio-DCA compounds were diluted in DMSO to a concentration of 2 M, and a working 0.25 M solution was subsequently prepared in phosphate buffered saline (PBS). The effect of the DCA or Thio-DCA compounds on cell viability was studied using

the CellTiter Blue™ viability assay (Promega, Madison, WI, USA). The cells were plated in quadruplicate in black, cell culture-treated, 96-well, optical, flat-bottom plates at a density of 8 × 10^4 cells/well. The studied compounds were added directly to the wells at different concentrations (1.25, 2.5, 5, 7.5, and 10 mM). After incubation in the presence of the drug for 96 h at 37 °C, 20% *v/v* CellTiter-Blue™ (Promega) reagent was added to the wells, followed by further incubation for 2 h at 37 °C. Finally, the fluorescence emission was directly read at 520 nm excitation and 610/30 nm emission in a Glomax® Multidetection System (Promega). Untreated cell controls (containing the same amount of DMSO as the treated wells) and wells with only reagents served as background controls and were run together with the treated cells. The absolute fluorescence was recorded in arbitrary units and the data were subsequently expressed as percentages relative to the untreated control cells. At least five independent repetitions of each data point were obtained. The significance of the differences in viability with the different treatments with respect to the controls was obtained using the Holm-Bonferroni and Holm-Sidak tests and the non-parametric Kolmogorov-Smirnov and Mann-Whitney tests in Origin 9.0 (OriginLab Co., Northampton, MA, USA).

S4. Supplementary Spectroscopy Figures of Dyes 1–4

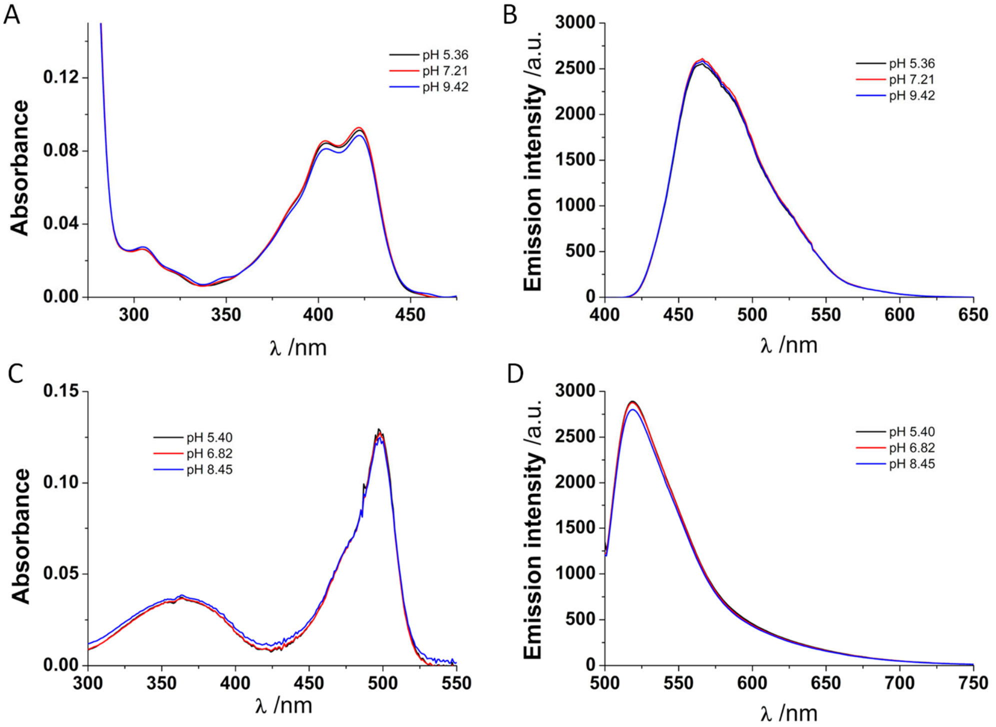

Figure S17. Absorption (A, C) and fluorescence emission (B, D) spectra of dyes 1 (A, B) and 4 (C, D) at different pH values. The excitation wavelength for the emission spectra was 375 nm for compound 1 and 495 nm for compound 4.

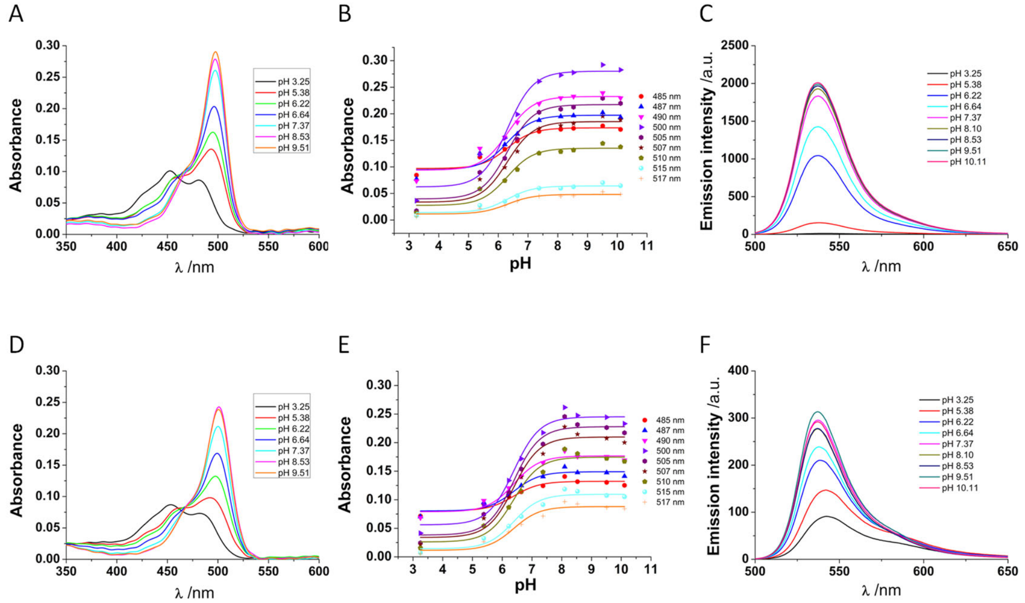

Figure S18. Absorption and fluorescence emission dependence with pH of dyes 2 (A–C) and 3 (D–F) in aqueous solution. A and D) Absorption spectra at different pH values of dyes 2 (A) and 3 (D). B and E) Global fittings of the *A vs* pH data to the general equilibrium equations [15,16] to obtain the ground state pK_a values of dyes 2 (B) and 3 (E). C and F) Fluorescence emission spectra (λ_{ex} = 490 nm) of dyes 2 (C) and 3 (F) in aqueous solution at different pH values.

S5. Viscosity Dependence of the Fluorescence Lifetime of Dye 3

Methanol:glycerine mixtures of different viscosity were prepared using different proportions ranging from 100% (*v*/*v*) methanol to 99% (*v*/*v*) glycerine. The density and viscosity values of the mixtures were experimentally measured as described in the supplementary methods. The faster deactivation of dye **3** in low-viscosity mixtures and the overall viscosity dependence of its ◎ values are related to the free rotation of the thiophene moiety with respect to the fluorophore xanthene core. Although this could serve as basis for the development of a viscosity sensor [17], the viscosity values to which dye **3** responds are lower than 1 mPa·s, which limits its use in biologically-relevant applications.

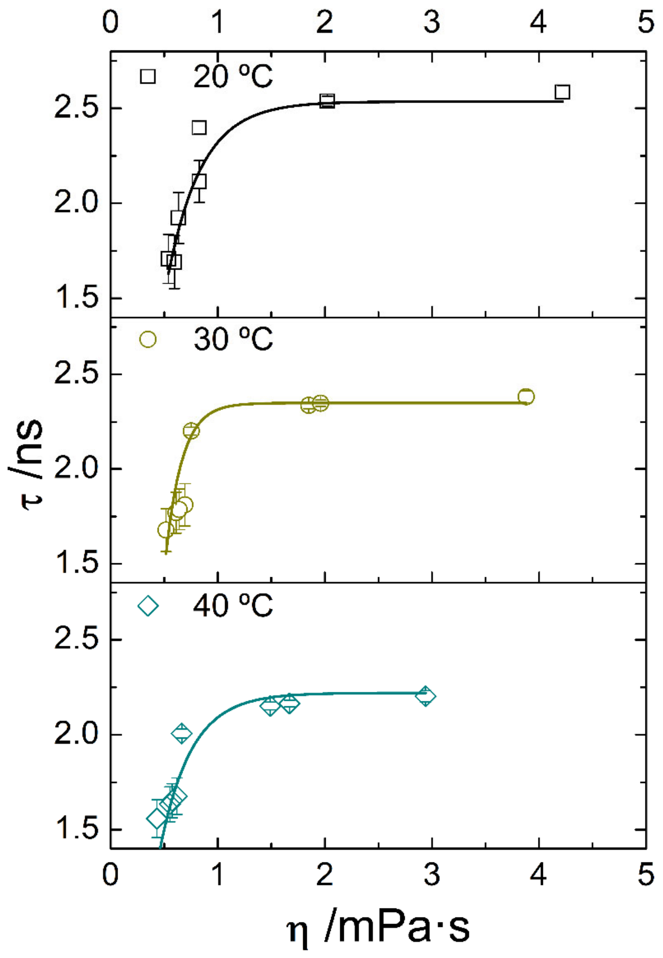

Figure S19. Average fluorescence lifetime, τ, of dye 3 in methanol:glycerine mixtures of different viscosity at 20, 30 and 40 °C.

S6. Photostability of Dyes 1–4

Fluorescence emission intensity was measured with λ_{ex} = 407 (for **1**) and 497 nm (for **2**–**4**). The excitation slit was open to 20 nm and the shutter was continuously open. The absorbance of the solutions was measured before the experiment, being in all cases lower than 0.1 at the λ_{ex}. Fluorescence emission spectra were collected every 5 min during 2 hours. Acridone **1** increased its emission by 16%, probably due to slow solubility. Xanthene derivative **2** was less photostable, showing a 57% decrease in intensity within the 2 h. In contrast, xanthene **3** and BODIPY **4** showed excellent photostability, exhibiting loss of fluorescence emission of 9% (**3**) and 3% (**4**), respectively. These results ensure that

the dyes are not drastically photodamaged during the time span of fluorescence imaging experiments (less than 1 h).

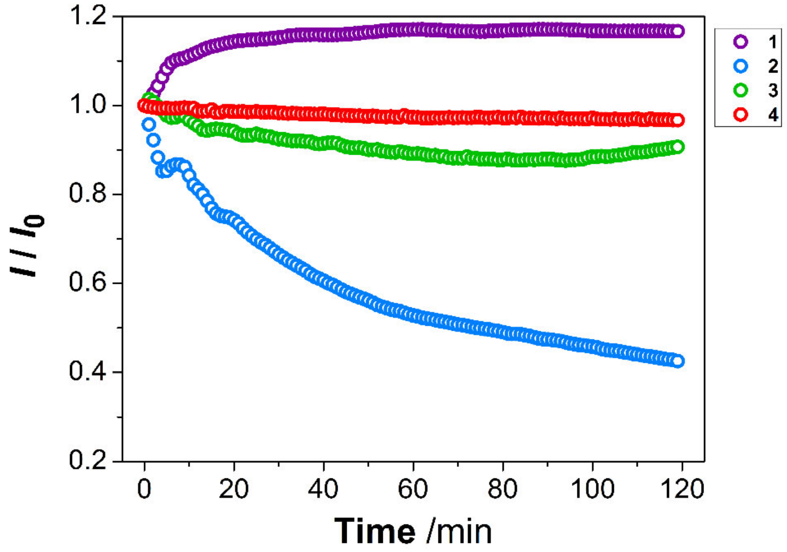

Figure S20. Photostability of dyes 1–4 during 2 h of continuous irradiation.

S7. Additional Colocalization Studies of Dyes 1–4 with MT

The following Supplementary Figures show additional results of the colocalization of the studied dyes, **1**–**4**, with the mitochondria tracker dye MT using either dual-color confocal or dual-color FLIM.

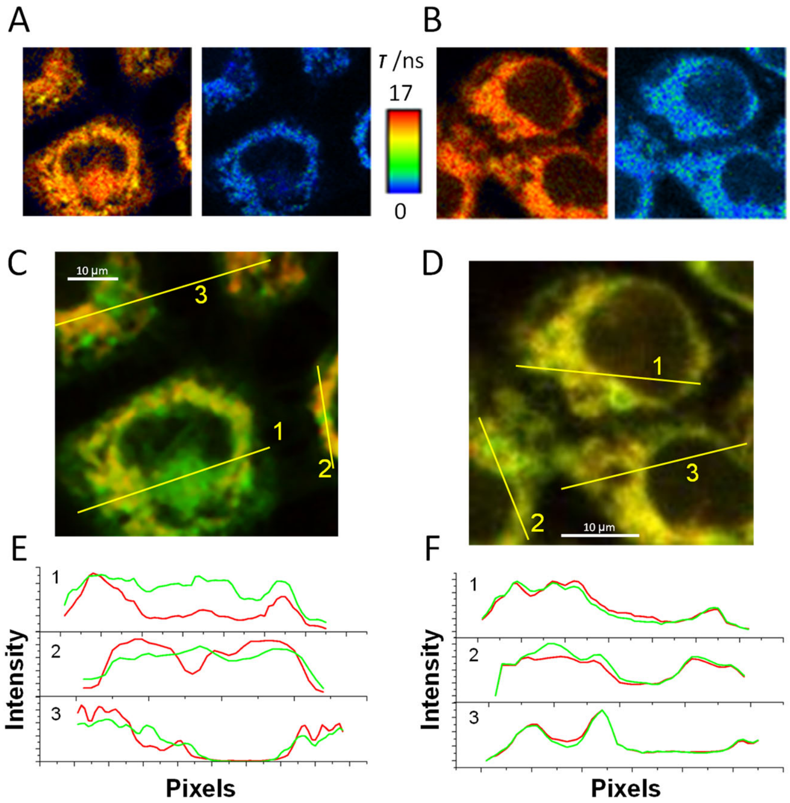

Figure S21. Representative dual-color FLIM images of compound 1 in 143B cells and $\varrho_0$206 cells after 20 min of incubation with MT. Panels (A) and (B) show FLIM images on a pseudo-color scale (between 0 and 17 ns) of the dye's detection channel (left) and the MT detection channel (right). These examples were performed on human osteosarcoma 143B cells (A) and $\varrho_0$206 cells (B). The latter are tumor cells depleted of mitochondrial DNA, thus displaying an extreme metabolic phenotype due to the absence of respiration [10]. Panels (C) and (D) show the colocalization images of 1 (green) and MT (red) in 143B cells (C) and $\varrho_0$206 cells (D). Scale bars represent 10 µm. Panels (E) and (F) show intensity traces in both channels for the depicted lines in images in panels C (for 143B cells) and D (for $\varrho_0$206 cells), respectively.

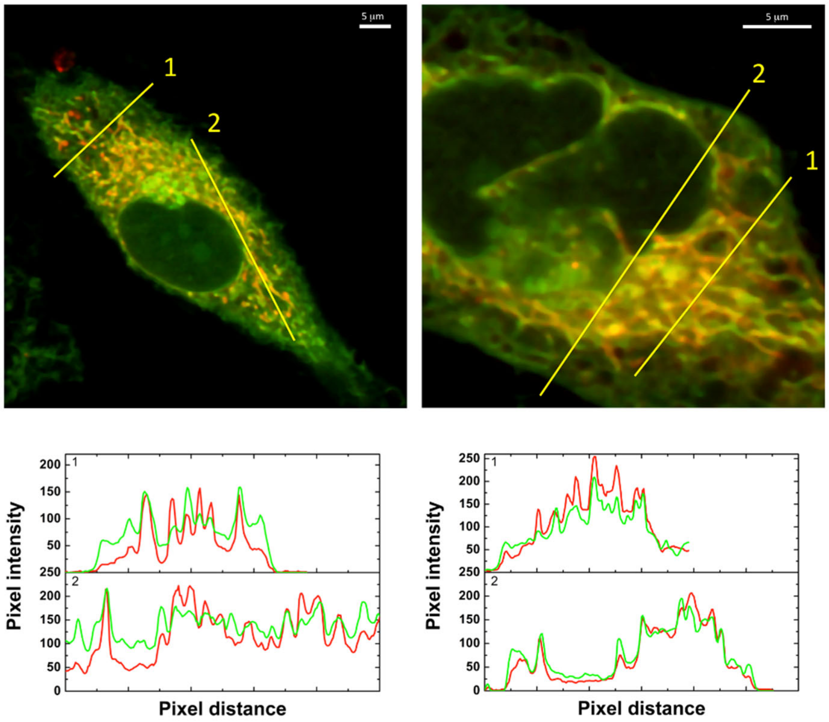

Figure S22. Representative dual-color, super-resolution optical fluctuation imaging (SOFI) of **1** (green channel) and MT (red channel) in formaldehyde-fixed HeLa cells, and intensity plots of the profile lines. Scale bars represent 5 µm.

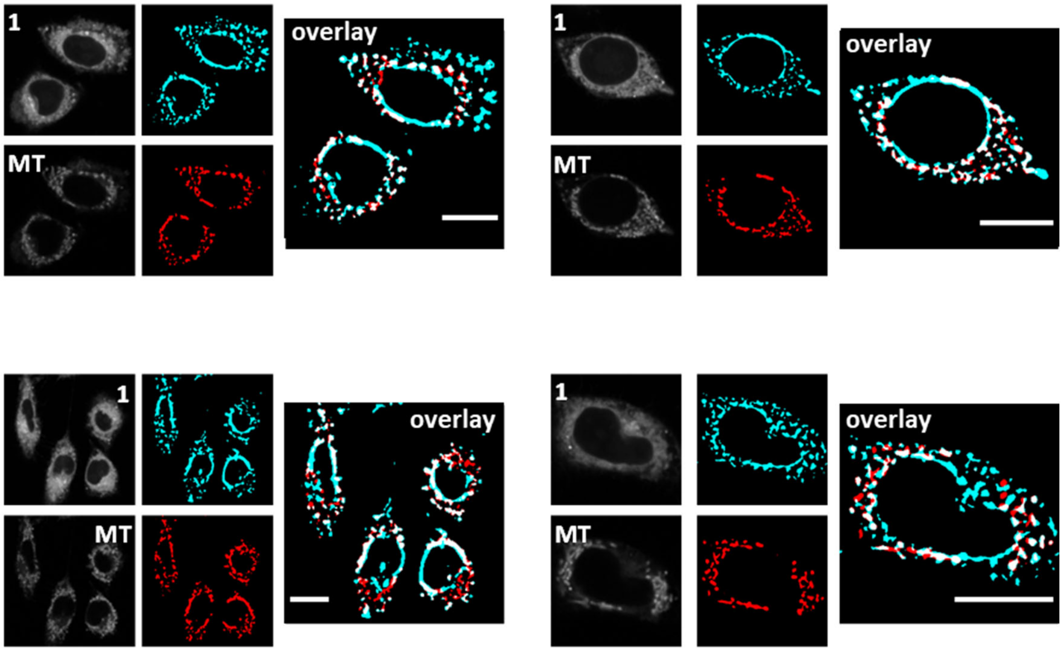

Figure S23. Mitochondrial localization of compound **1** in 143B cells, after 20 min of incubation with MT, from dual-color FLIM images. Left panels represent the raw intensity images in the **1** channel and the MT channel. Central panels represent the selected region of interest, in cyan for compound **1** and red for MT. Rightmost panels are the overlaid images, with colocalized pixels represented in white color. Scale bars represent 10 µm.

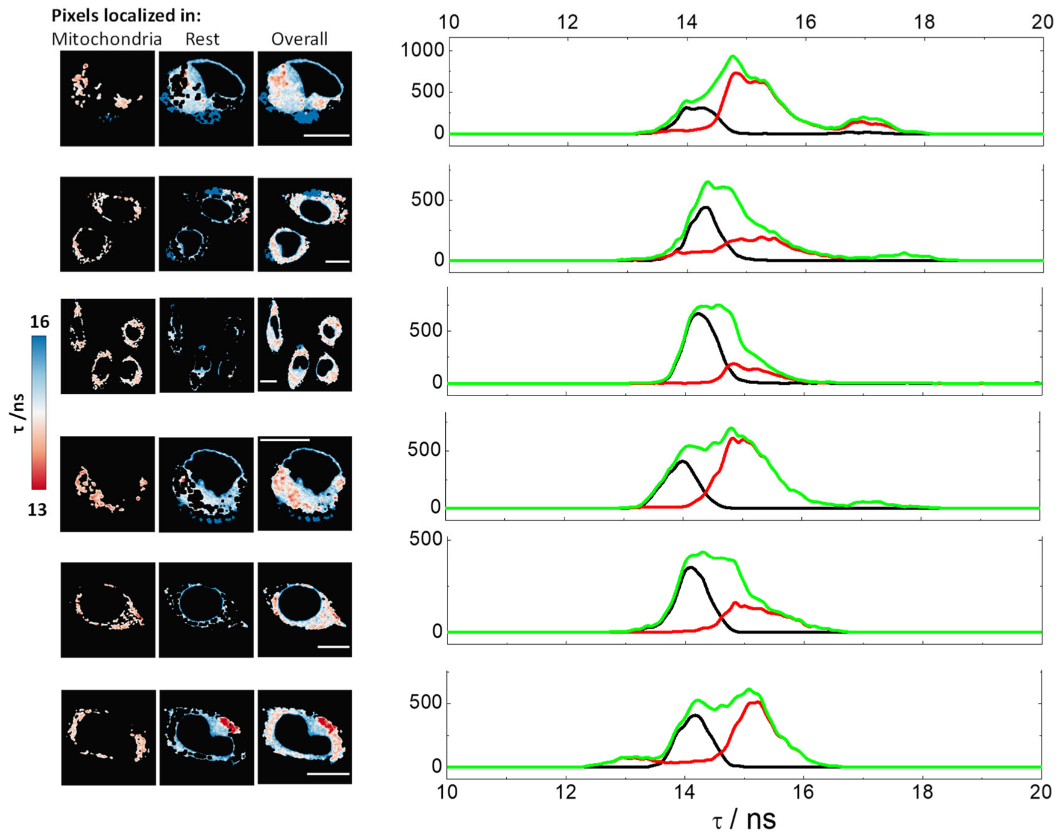

Figure S24. FLIM imaging of compound **1** in 143B cells. The images show the fluorescence lifetime of **1** depicted on a pseudocolor scale between 13 and 16 ns. The leftmost column of images shows colocalized pixels with mitochondria. The central column of images shows non colocalized pixels. The rightmost column of images shows the overall images. Scale bars represent 10 μm. The plots on the right panels represent the pixel distribution of fluorescence lifetime of **1** in each image, localized in mitochondria (black lines) or in other cellular subcompartments (red lines), and the overall lifetime distribution (green lines). Given that the acridone core has a lifetime that depends on the polarity of the microenvironment [18], these data clearly show that the mitochondria matrix is a less polar environment than cellular cytoplasm.

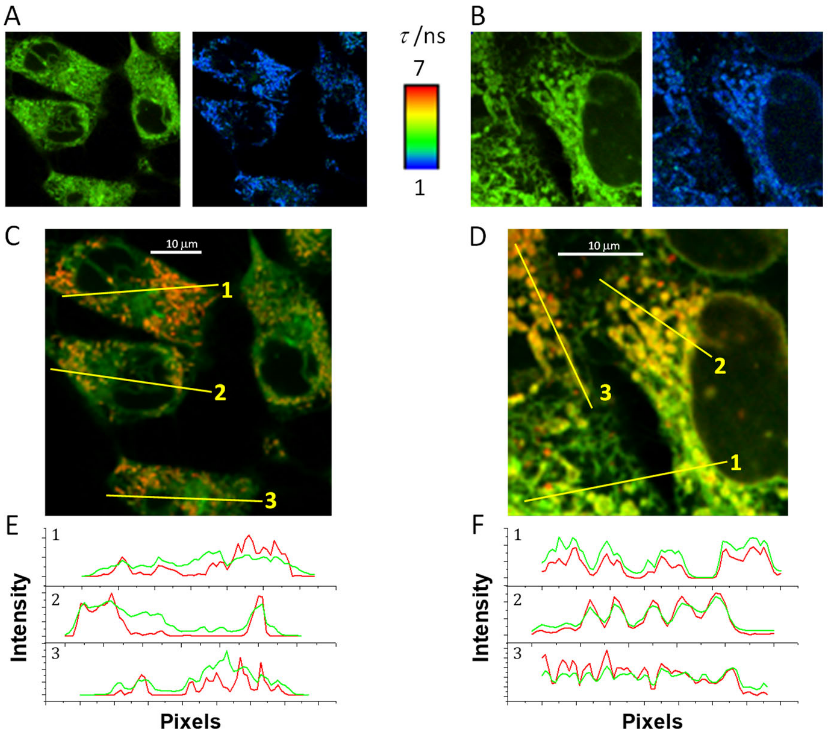

Figure S25. Representative dual-color FLIM images of compound **2** in 143B (**A**, **C**, and **E**) and ρ0206 cells (**B**, **D**, and **F**), after 20 min of incubation with MT. In the FLIM images (**A** and **B**), the dye's detection channel (left) and the MT detection channel (right) are shown separately. The colocalization images (**C** and **D**) of the dye (green) and MT (red) are also shown. Intensity traces in both channels (**E** and **F**) are shown for the depicted lines (marked as 1, 2 and 3) in the colocalization panels. Scale bars represent 10 µm.

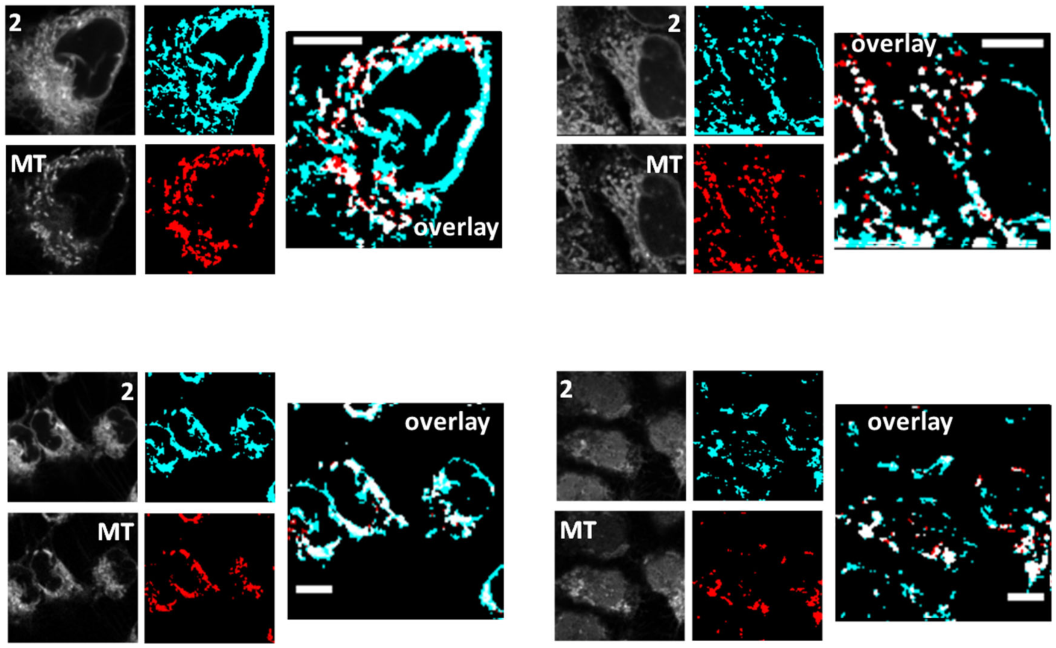

Figure S26. Mitochondrial localization of compound **2** in ρ0206 cells, after 20 min of incubation with MT, from dual-color FLIM images. Left panels represent the raw intensity images in the **2** channel and the MT channel. Central panels represent

the selected region of interest, in cyan for compound **2** and red for MT. Rightmost panels are the overlaid images, with colocalized pixels represented in white color. Scale bars represent 10 µm.

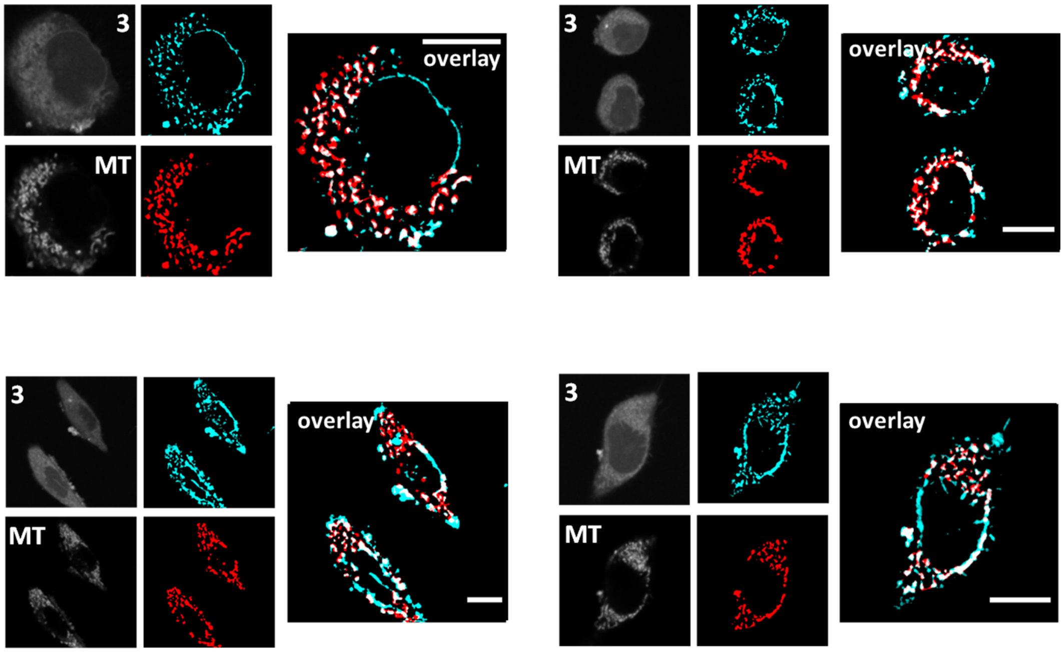

Figure S27. Mitochondrial localization of compound **3** in MDA-MB-231 cells, after 20 min of incubation with MT, from dual-color FLIM images. Left panels represent the raw intensity images in the **3** channel and the MT channel. Central panels represent the selected region of interest, in cyan for compound **3** and red for MT. Rightmost panels are the overlaid images, with colocalized pixels represented in white color. Scale bars represent 10 µm.

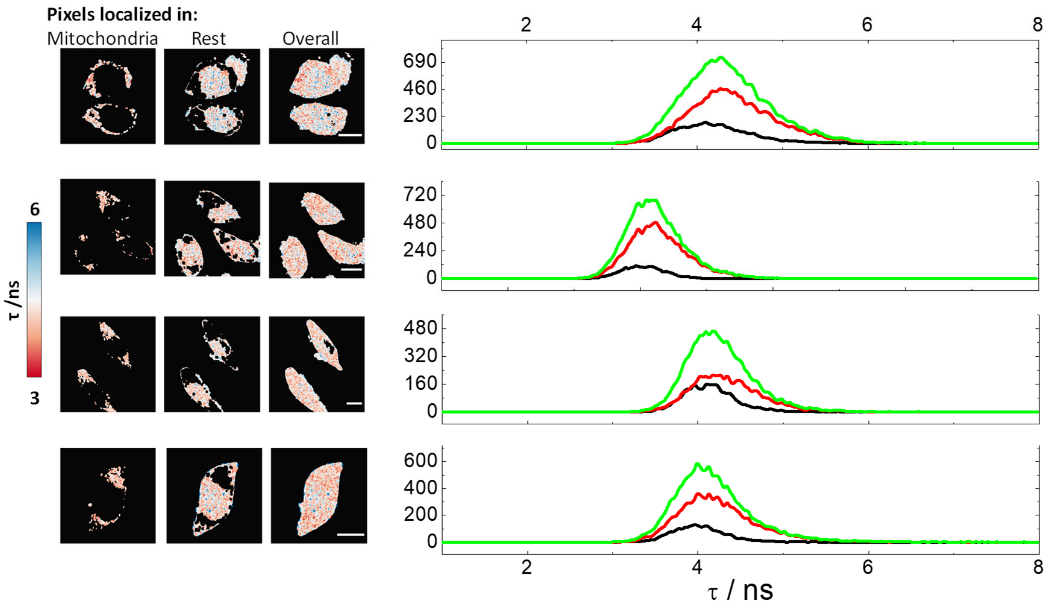

Figure S28. FLIM imaging of compound **3** in MDA-MB-231 cells. The images show the fluorescence lifetime of **3** depicted on a pseudocolor scale between 3 and 6 ns. The leftmost column of images shows colocalized pixels with mitochondria. The central column of images shows non colocalized pixels. The rightmost column of images shows the overall images. Scale bars represent 10 µm. The plots on the right panels represent the pixel distribution of fluorescence lifetime of **3** in each image, localized in mitochondria (black lines) or in other cellular subcompartments (red lines), and the overall lifetime distribution (green lines).

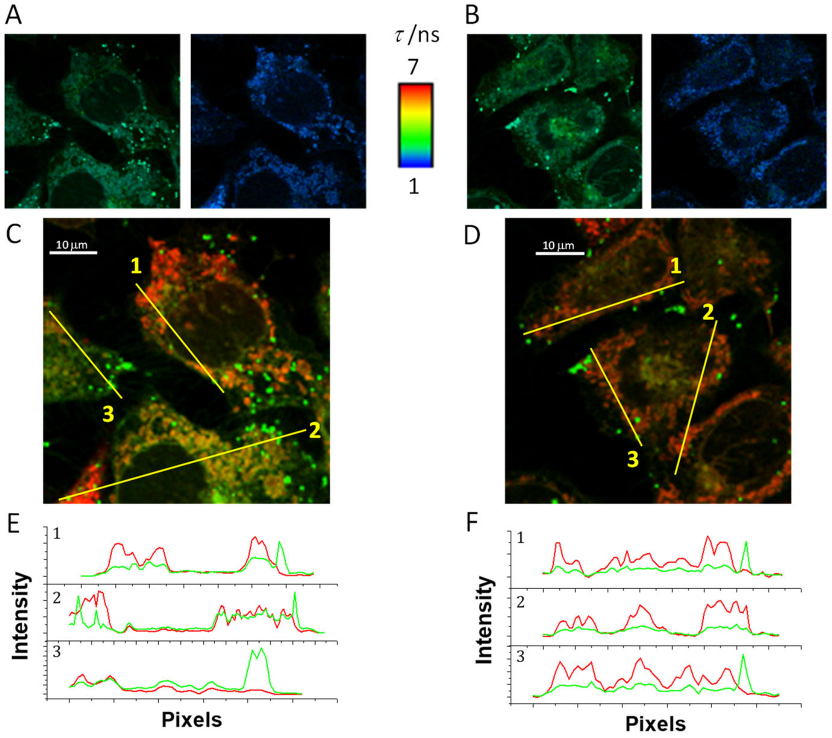

Figure S29. Representative dual-color FLIM images of compound **4** in 143B (**A**, **C**, and **E**) and ϱ0206 cells (**B**, **D**, and **F**), after 20 min of incubation with MT. In the FLIM images (A and B), the dye's detection channel (left) and the MT detection channel (right) are shown separately. The colocalization images (C and D) of the dye (green) and MT (red) are also shown. Intensity traces in both channels (E and F) are shown for the depicted lines in the colocalization panels. Scale bars represent 10 µm.

Table S2. Pearson's correlation coefficient (PCC) and Manders' colocalization coefficient (MCC) values for the colocalization of dyes **1–4** with the mitochondria tracker MT.[a].

Compound.	PCC	MCC Dye Channel	MCC MT Channel
**1**	0.59 ± 0.10	0.46 ± 0.14	0.64 ± 0.10
**2**	0.69 ± 0.07	0.64 ± 0.14	0.73 ± 0.14
**3**	0.58 ± 0.12	0.58 ± 0.15	0.66 ± 0.12
**4**	0.67 ± 0.04	0.46 ± 0.06	0.36 ± 0.12

[a] All the values are averages from, at least, five different images. Errors are reported as standard deviation.

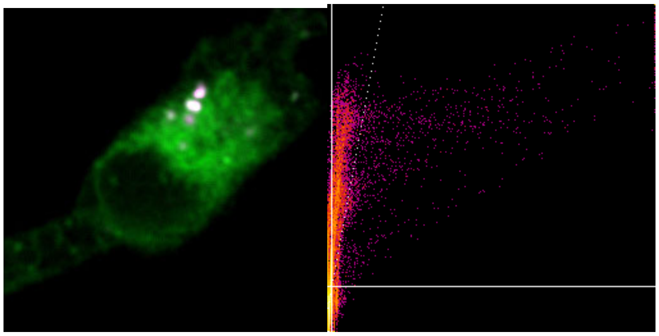

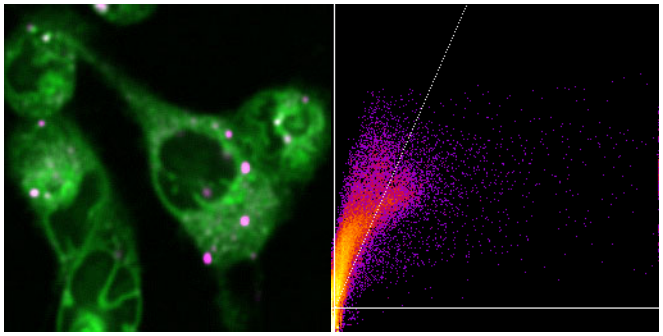

Figure S30. Representative dual-color fluorescence images of compound **1** (green) and the MT tracker (magenta) in 143B cells after 20 min of incubation with BAM15. Scatter plots represent the correlation of the intensity values in the green channel *vs* the red channel. Scale bars represent 10 µm.

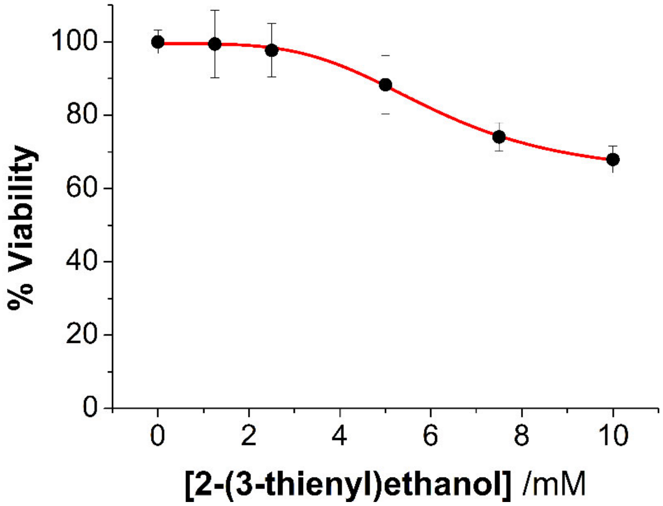

Figure S31. Cell viability of SKBR3 breast cancer tumor cells treated with 2-(3-thienyl)ethanol at different doses. Error bars represent standard deviations. Line represents the fit to a dose-response function.

References

1. Chen,C.; Hong, S.H. Selective catalytic sp3 C-O bond cleavage with C-N bond formation in 3-alkoxy-1-propanols. *Org. Lett.*, **2012**, *14*, 2992-2995, doi:10.1021/ol3009842.
2. Huynh, H.V.; Chew, Y.X. Synthesis, structural characterization and catalytic activity of a palladium(II) complex bearing a new ditopic thiophene-N-heterocyclic carbene ligand. *Inorg. Chim. Acta*, **2010**, *363*, 1979-1983, doi:10.1016/j.ica.2009.02.035.
3. Smith, J.A.; West, R.M.; Allen, M. Acridones and quinacridones: Novel fluorophores for fluorescence lifetime studies. *J Fluoresc.*, **2004**, *14*, 151-171, doi:.
4. Gonzalez-Garcia, M.C.; Herrero-Foncubierta, P.; Castro, S.; Resa, S. Alvarez-Pez, J.M. Miguel D. et al., Coupled Excited-State Dynamics in N-Substituted 2-Methoxy-9-Acridones. *Front. Chem.*, **2019**, *7*, 129 (1-13), doi:10.3389/fchem.2019.00129.
5. Teeuwen, R.L.; van Berkel, S.S.; van Dulmen, T.H.; Schoffelen, S.; Meeuwissen, S.A. Zuilhof H. et al., "Clickable" elastins: Elastin-like polypeptides functionalized with azide or alkyne groups. *Chem. Commun.*, **2009**, 4022-4024, doi:10.1039/b903903a.
6. Choi, S.H.; Kim, K.; Lee, J.; Do, Y.; Churchill, D.G. X-ray diffraction, DFT, and spectroscopic study of N,N′-difluoroboryl-5-(2-thienyl)dipyrrin and fluorescence studies of related dipyrromethanes, dipyrrins and BF2-dipyrrins and DFT conformational study of 5-(2-thienyl)dipyrrin. *J Chem. Crystallogr.*, **2007**, *37*, 315-331, doi:10.1007/s10870-006-9126-0.
7. Müller, B.K.; Zaychikov, E.; Bräuchle, C.; Lamb, D.C. Pulsed Interleaved Excitation. *Biophys. J.*, **2005**, *89*, 3508-3522, doi:.
8. Schindelin, J.; Arganda-Carreras, I.; Frise, E.; Kaynig, V.; Longair, M.; Pietzsch, T. et al., Fiji: An open-source platform for biological-image analysis. *Nat. Meth.*, **2012**, *9*, 676-682, doi:10.1038/nmeth.2019.
9. Schindelin, J.; Rueden, C.T.; Hiner, M.C.; Eliceiri, K.W. The ImageJ ecosystem: An open platform for biomedical image analysis. *Mol. Reprod. Dev.*, **2015**, *82*, 518-529, doi:10.1002/mrd.22489.

10. King, M.; Attardi, G. Human cells lacking mtDNA: Repopulation with exogenous mitochondria by complementation. *Science*, **1989**, *246*, 500-503, doi:10.1126/science.2814477.
11. Herrero-Foncubierta, P. M.; González-García, d.C. Resa, S.; Paredes, J.M.; Ripoll, C.; Girón M.D. et al., Simple and non-charged long-lived fluorescent intracellular organelle trackers. *Dyes and Pigments*, **2020**, *183*, 108649, doi:10.1016/j.dyepig.2020.108649.
12. Denofrio, M.P;. Rasse-Suriani, F.A.O.; Paredes, J.M.; Fassetta, F.; Crovetto, L.; Giron, M.D. et al., N-Methyl-β-carboline alkaloids: Structure-dependent photosensitizing properties and localization in subcellular domains. *Org. Biomol. Chem.*, **2020**, *18*, 6519-6530, doi:10.1039/D0OB01122C.
13. Dunn, K.W.; Kamocka, M.M.; McDonald, J.H. A practical guide to evaluating colocalization in biological microscopy. *Am. J. Physiol. Cell Physiol.*, **2011**, *300*, C723-C42, doi:10.1152/ajpcell.00462.2010.
14. Bolte, S.; Cordelières, F.P. A guided tour into subcellular colocalization analysis in light microscopy. *J. Microsc.*, **2006**, *224*, 213-232, doi:10.1111/j.1365-2818.2006.01706.x.
15. Orte, A.; Talavera, E.M. Maçanita, A.L.; Orte, J.C.; Alvarez-Pez, J.M. Three-State 2',7'-Difluorofluorescein Excited-State Proton Transfer Reactions in Moderately Acidic and Very Acidic Media. *J. Phys. Chem. A.*, **2005**, *109*, 8705-8718, doi:10.1021/jp051264g.
16. Orte, A.; Crovetto, L.; Talavera, E.M.; Boens, N.; Alvarez-Pez, J.M. Absorption and Emission Study of 2',7'-Difluorofluorescein and Its Excited-State Buffer-Mediated Proton Exchange Reactions. *J. Phys. Chem. A.*, **2005**, *109*, 734-747, doi:10.1021/jp046786v.
17. Miguel, D.; Morcillo, S.P.; Martin-Lasanta, A.; Fuentes, N.; Martinez-Fernandez, L. Corral, I. et al., Development of a New Dual Polarity and Viscosity Probe Based on the Foldamer Concept. *Org. Lett.*, **2015**, *17*, 2844-2847, doi:10.1021/acs.orglett.5b01275.
18. Gonzalez-Garcia, M.C.; Herrero-Foncubierta, P.; Garcia-Fernandez, E.; Orte, A. Building Accurate Intracellular Polarity Maps through Multiparametric Microscopy. *Methods Protoc.*, **2020**, *3*, 78, doi:10.3390/mps3040078.
